# Analysis of Familial Hemophagocytic Lymphohistiocytosis Type 4 (FHL-4) Mutant Proteins Reveals that S-Acylation Is Required for the Function of Syntaxin 11 in Natural Killer Cells

**DOI:** 10.1371/journal.pone.0098900

**Published:** 2014-06-09

**Authors:** Andrew L. Hellewell, Ombretta Foresti, Nicola Gover, Morwenna Y. Porter, Eric W. Hewitt

**Affiliations:** School of Molecular and Cellular Biology and Astbury Centre for Structural Molecular Biology, University of Leeds, Leeds, United Kingdom; Cambridge University, United Kingdom

## Abstract

Natural killer (NK) cell secretory lysosome exocytosis and cytotoxicity are impaired in familial hemophagocytic lymphohistiocytosis type 4 (FHL-4), a disorder caused by mutations in the gene encoding the SNARE protein syntaxin 11. We show that syntaxin 11 binds to SNAP23 in NK cells and that this interaction is reduced by FHL-4 truncation and frameshift mutation proteins that delete all or part of the SNARE domain of syntaxin 11. In contrast the FHL-4 mutant proteins bound to the Sec-1/Munc18-like (SM) protein Munc18-2. We demonstrate that the C-terminal cysteine rich region of syntaxin 11, which is deleted in the FHL-4 mutants, is S-acylated. This posttranslational modification is required for the membrane association of syntaxin 11 and for its polarization to the immunological synapse in NK cells conjugated to target cells. Moreover, we show that Munc18-2 is recruited by syntaxin 11 to intracellular membranes in resting NK cells and to the immunological synapse in activated NK cells. This recruitment of Munc18-2 is abolished by deletion of the C-terminal cysteine rich region of syntaxin 11. These results suggest a pivotal role for S-acylation in the function of syntaxin 11 in NK cells.

## Introduction

Natural killer (NK) cells are specialized immune cells that eliminate pathogen infected and tumorigenic cells [1]. Target cell killing is mediated by the secretion of perforin and granzymes, which are stored within the secretory lysosomes of NK cells [2]–[4]. The recognition of a target cell induces the formation of an activating immunological synapse at the contact site of the two cells [4], [5]. Secretory lysosomes are polarized towards the immunological synapse, where they fuse with the plasma membrane releasing their cytotoxic contents [4]–[6]. The pore forming protein perforin then facilitates the entry of pro-apoptopic granzymes into the target cell cytoplasm resulting in cell death [2], [3].

NK cell cytotoxicity is severely impaired in the hematological disorder familial hemophagocytic lymphohistiocytosis (FHL). Subtypes 4 (FHL-4) and 5 (FHL-5) are caused by the mutation of genes encoding syntaxin 11 and Munc18-2 respectively [7]–[16]. Analysis of NK cells isolated from subjects with FHL-4 and FHL-5 revealed a defect in secretory lysosome exocytosis [8], [10]–[16]. In these cells recognition of a target cell causes secretory lysosomes to polarize to the activating immunological synapse, but they are unable to fuse with the NK cell plasma membrane and cannot release their contents [8], [10]–[16].

Syntaxin 11 is a soluble N-ethylmaleimide (NEM)-sensitive factor attachment protein receptor (SNARE), a class of proteins that catalyse membrane fusion reactions by forming trans-SNARE complexes that bridge opposing membranes [17], [18]. It is abundant in the immune system and is expressed by B lymphocytes, cytotoxic T lymphocytes (CTLs), dendritic cells, mast cells, monocytes, macrophages, NK cells and neutrophils [19]–[26]. In addition to a role in secretory lysosome exocytosis in NK cells, syntaxin 11 has been reported to be required for the exocytosis of secretory organelles in CTLs, neutrophils and platelets [26], [27], whereas in macrophages it inhibits phagocytosis and regulates late endosome-lysosome fusion [21], [28]. Despite its role in exocytosis of secretory lysosomes by NK cells, syntaxin 11 does not co-localize with secretory lysosomes in resting NK cells [20],[29], but it is polarized to the immunological synapse when NK cells are activated by conjugation to target cells [29]. Furthermore, syntaxin 11 interacts with Munc18-2 [12], [13], [23], [30], a member of the Sec-1/Munc18-like (SM) family of proteins whose members regulate SNARE-mediated membrane fusion reactions [18]. SM proteins also chaperone syntaxins, regulating the level and localization of these SNAREs [31]–[36]. This chaperone function is evident in FHL-5, in which mutations in Munc18-2 result in a pronounced reduction in the level of syntaxin 11 [12], . In contrast, how mutations associated with FHL-4 result in the loss of function of syntaxin 11 in NK cells is poorly understood.

Herein we dissect the molecular basis for FHL-4 by examining how disease-associated mutations affect the interaction of syntaxin 11 with other proteins and cellular membranes. FHL-4 deletion and frameshift mutations result in the abrogation of secretory lysosome exocytosis and the consequent loss of NK cell cytotoxicity [7]–[11]. We show that these FHL-4 mutations have differential effects on SNARE binding by syntaxin 11, but the FHL-4 mutant proteins retain a Munc18-2 binding site. Moreover, syntaxin 11 is S-acylated in NK cells and this is dependent on the C-terminal cysteine rich region, which is deleted in all of the FHL-4 mutants characterized. This posttranslational modification is required for the membrane association of syntaxin 11 and for the polarization of this protein to the immunological synapse. We also show that syntaxin 11 recruits Munc18-2 to intracellular membranes and that this is dependent on the cysteine rich region of syntaxin 11. Together these findings demonstrate an important role for S-acylation in the function of syntaxin 11 in NK cells.

## Materials and Methods

### Antibodies

The following antibodies were used: rabbit anti-syntaxin 11 (Proteintech Group Inc), mouse polyclonal anti-syntaxin 11 (Abcam), rabbit anti-synaptosomal-associated protein, 23 kDa (SNAP23, Synaptic Systems), mouse anti-glyceraldehyde 3-phosphate dehydrogenase (GAPDH, clone 6C5, Abcam), rabbit anti-calreticulin (Calbiochem), mouse anti-Myc epitope tag (clone 9E10, Sigma Aldrich) and rabbit anti-calnexin (Stressgen).

### DNA constructs

#### (i) Syntaxin 11 green fluorescent (GFP) fusions

A construct encoding a GFP fusion of the wild type human syntaxin 11 (pEGFP-C3-syntaxin 11) was generated by PCR through extension of the truncated syntaxin 11 open reading frame in pCMV-Tag3a-syntaxin 11 (a kind gift from Dr R. Prekeris) using the oligonucleotides 5′syn11 and 3′Syn11, the resultant PCR product was then inserted into pEGFP-C3 (Clontech) at the *Xho* I and *Eco* RI sites. A GFP fusion of the syntaxin 11-Q268X mutant was generated by PCR using pEGFP-C3-syntaxin 11 as a template and the oligonucleotides 5′Syn11 and 3′Q268X, the resultant PCR product was cloned into pEGFP-C3 at the *Xho* I and *Eco* RI sites. The GFP fusion of the N-terminal deletion syntaxin 11ΔN24 was cloned by amplifying pEGFP-C3-syntaxin 11 with the oligonucleotides 5′Syn11ΔN24 and 3′Syn11 and cloning the resultant product into pEGFP-C3. The cysteine mutant syntaxin 11C5A was cloned by amplifying pEGFP-C3-syntaxin 11 with the primers 5′Syn11 and 3′Syn11C5A and cloning the resultant product into the *Xho* I and *Eco* RI sites of pEGFP-C3. Oligonucleotide sequences are provided in Table 1.

**Table 1:** Oligonucleotide sequences.

Oligonucleotide	Sequence
5′Syn11	ATATCTCGAGACCATGAAAGACCGGCTAGCAGAACTTCTGGACTTGTCC
3′Syn11	ATATGAATTCTACTTGAGGCAGGGACAGCAGAAGCAGCAGAGGGTCCGGCAGGGGTTCTTCTCCTCGTACTGCACGCCTTCCGCACCTGCGCCTTGGC
3′Q268X	ATATATGAATTCTCTAGACTACACGGCCTTCCGCACCTGC
5′Syn11ΔN24	ATATCTCGAGGACTCGCCCCACGAGGACATC
Syn11C5A	GGAATTCTACTTGAGGGCGGGAGCGGCGAAGGCGGCGAGGGTCCGGCAGGG
5′Syn11	ATATGAATTCAAAGACCGGCTAGCAGAACTT
3′Syn11GST	ATATCTCGAGCTACTTGAGGCAGGGACAGCAG
3′Syn11Q268XGST	ATATCTCGAGTCACACGGCCTTCCGCACCTGCGC
L194	TTTCCGAGAACTTGCCGACGTGAAGGGCGC
V124	CCAGCACGGCCCGCACTCGGCTGGGCATTTCGCGGGCGCAGTACA
T37	ACATCGTGTTCGAGAGGACCACATCCTGGA
E25	CCAGACGGGGACGATTGAGTTTGACTCGCCC
Q230FsR	CAGCACCGCCATCTGCCAAGAAGAGCTCGTG
3′Q230GST	ATATGAATTCTCATCGGACGGTATCAACTAA
Q230FsF	CACGAGCTCTTCTTGGCAGATGGCGGTGCTG
Munc18NT-F1	ATTTCTGAAGAAGATCTGGCCGGCCTGGGAGGATCGGCGCCCTCGGGGCTGAAGGC
Munc18NT-F2	TCTCTCGAATTCGCCGCCACCATGGAACAA AAACTTATTTCTGAAGAAGATCTGGCCGGC
Munc18NT-R2	GAGAGAGGATCCTCAGGGCAGGGCAATGTCCTCC

#### (ii) Syntaxin 11 Glutathione S-transferase (GST) fusions

A GST fusion construct of the wild type human syntaxin 11 (pGEX4T.1-syntaxin 11) was cloned by PCR amplification of the syntaxin 11 sequence using pEGFP-C3-syntaxin 11 as a temple and the oligonucleotides 5′Syn11GST and 3′Syn11GST. The resultant PCR product was inserted into the *Xho* I and *Eco* RI sites of pGEX4T.1 (GE Life Sciences). The cloning of a GST fusion of syntaxin 11 Q268X was identical to that of the wild type sequence except that the oligonucleotide 3′Syn11Q268XGST was used instead of 3′Syn11GST. Constructs encoding GST fusions of the syntaxin 11 mutants L194fsX2, V124fsX60, T37fsX62 and E25X were generated using the QuikChange site-directed mutagenesis kit (Stratagene) as per the manufacturer's instructions using pGEX4T.1-syntaxin 11 as a template and the sense oligonucleotides L194, V124, T37 and E25 respectively; the antisense oligonucleotides were the exact complement. The GST fusion of the mutant Q230fsX125, was cloned using a two-step PCR procedure. The syntaxin 11 cDNA sequence (MGC clone 5176646) was used as a template and amplified with either 5′Syn11GST and Q230FsR or with 3′Q230GST and Q230FsR oligonucleotides. The two PCR products were gel purified, mixed together and used as the template in a second stage PCR reaction with the oligonucleotides 5′Syn11GST and 3′Q230GST, the resultant PCR product was cloned into pGEX4T.1 at the *Xho* I and *Eco* RI sites. Oligonucleotide sequences are provided in Table 1.

#### (iii) Munc18-2 constructs

To generate a construct encoding human Munc18-2 tagged at the N-terminus with a Myc-epitope, a two step PCR procedure was used. The primers Munc18NT-F1 and Munc18NT R1b were used in step 1 to amplify the Munc18-2 human cDNA clone (MGC clone 71251). The gel purified PCR product was then used as the template in a second PCR reaction and amplified with the primers Munc18NT-F2 and Munc18NT R2. The resultant PCR product was then cloned into pcDNA3.1Pac(-) using *Eco* R1 and *Bam* H1 to generate pCDNA3.1Pac(-)-Myc-Munc18-2. To generate a mCherry fusion, the Munc18-2 open reading frame was cut out of pcDNA3.1Pac(-)-Myc-Munc18-2 and inserted into the pmCherry-C2 vector (Clontech) using *Eco* R1 and *Bam* H1. Oligonucleotide sequences are provided in Table 1.

### Culture and transfection of cells

HeLa-M and YTS cells were cultured as described previously [20], [37]. 721.221 cell lines were maintained in RPMI 1640 media supplemented with 10% (v/v) fetal bovine serum (FBS). HeLa-M cells were transfected with Lipofectamine 2000 (Invitogen) as per the manufacturer's instructions. YTS NK cells were transfected by nucleofection (Amaxa). Briefly, 5×10^6^ YTS cells were resuspended in 100 µl nucleofector solution R, to which 5 µg plasmid DNA was added. Cells were nucleofected using program X-01 and mixed immediately with 500 µl RPMI 1640 supplemented with 20% (v/v) FBS. After 5 min at 37°C, 5% CO_2_, cells were plated out in 2 ml of growth medium at 37°C, 5% CO_2_, an additional 2 ml of growth medium was added 1 h post nucleofection.

### Immunoprecipitation

10^7^ YTS cells per immunoprecipitation were incubated in the presence or absence of 100 mM NEM for 30 min prior to lysis. Cells were lysed into NET buffer [50 mM Tris pH 7.5, 150 mM NaCl, 1 mM ethylenediaminetetraacetic acid (EDTA), 0.1% Nonidet-P40, 0.25% gelatin and complete protease inhibitors (Roche Applied Science)] for 30 min on ice before the lysate was centrifuged at 13,000 g for 10 min at 4°C in a microcentrifuge. The supernatant was precleared by incubation for 1 h with protein G sepharose (Sigma Aldrich) and centrifuged at 13,000 g for 1 min. Mouse syntaxin 11 antibody was added to the supernatant and incubated on ice for 2 h, protein G-sepharose was then added and the samples rotated for 2 h at 4°C. The beads were washed 4 times with NET buffer and bound proteins eluted by boiling in Laemmli sample buffer.

### GST Pulldowns

BL21(DE3) pLys was transformed with each GST expression plasmid, cultured in 100 ml Luria-Bertani medium containing 100 µg/ml ampicillin (100 µg/ml) and grown at 37°C with shaking at 200 rpm to an OD_600_ of 0.6. The culture was then induced with 1 mM isopropyl-β-d-thiogalactopyranoside and grown for a further 4 h. The cells were harvested by centrifugation and resuspended into 2 ml STEP buffer [10 mM Tris pH8.0, 150 mM NaCl 1 mM EDTA, 1 mM dithiothreitol, 1 mM phenylmethanesulfonyl fluoride] with 0.25% (w/v) sarkosyl on ice. The bacterial suspension was then sonicated on ice and Triton X-100 added to a final concentration of 0.5% (v/v). Cellular debris was pelleted by centrifugation in a microcentrifuge for 10 min at 13,000 g. GST fusion proteins were purified from the supernatant with glutathione-Sepharose 4B affinity beads as specified by the manufacturer (Pharmacia Biotech). 5×10^6^ HeLa-M cells transfected with pcDNA3.1(-)-Myc-Munc18-2 were used per pulldown. 48 h post transfection, the cells were lysed on ice in STEP buffer with 0.5% Triton X-100 and centrifuged at 13,000 g in a microcentrifuge for 10 min. The supernatant was added to 50 µg of each GST-fusion bound to beads and incubated at 4°C with rotation for 2 h. The beads were washed 4 times with STEP buffer, with 0.5% Triton X-100, and bound proteins eluted by boiling in Laemmli sample buffer.

### Flow cytometric analysis of syntaxin 11 expression

HeLa-M cells were transfected with GFP-syntaxin 11 expression constructs and co-transfected with either the control plasmid pcDNA3.1(-)Pac or pcDNA3.1(-)Pac-MycMunc18-2. Each transfection was performed in triplicate. 48 h post-transfection the cells were washed once with phosphate buffered saline 0.1% bovine serum albumin and resuspended into PBS 0.1% BSA. The cells were analysed on a BD-LSRFortessa flow cytometer (Becton Dickinson), the cell population was gated to exclude debris, 10,000 gated events were analyzed and the mean fluorescence value of the total cell population was determined.

### Membrane Fractionation

10^7^ YTS cells were resuspended into homogenization buffer [25 mM 4-(2-hydroxyethyl)-1-piperazineethanesulfonic acid pH 7.5, 250 mM sucrose, 1 mM EDTA, complete protease inhibitors), homogenized using a ball bearing homogeniser (Isobiotec) with a 10 µm clearance and centrifuged at 400 g for 5 min at 4°C in a microcentrifuge. The resultant postnuclear supernatant was centrifuged at 50,000 g for 30 min at 4°C in an S100-AT3 rotor (Sorvall) to separate the cytosol (supernatant) from the membrane fraction (pellet).

### Live Cell Confocal Microscopy

5 h post-transfection, YTS cells were centrifuged on Lymphoprep (Axis Shield) at 850 g for 20 min in a bench top centrifuge to remove cellular debris, washed in cell media and allowed to recover for 1 h by incubation in growth medium at 37°C. To visualize secretory lysosomes the YTS cells were preloaded with 10 nM LysoTracker Red or LysoTracker Green (Molecular Probes). Cells were washed in phenol red free RPMI 1640 media supplemented with 10% FBS, 5×10^5^ cells were resuspended into 2 ml of the same medium and incubated in 35 mm glass bottomed dishes (Ibidi). In order to visualize conjugated cells, 2.5×10^5^ YTS cells were incubated in the glass bottomed dishes at 37°C for 15 min with 2.5×10^5^ 721.221 target cells that had been preloaded with 6 µM Cell Trace Far Red (Molecular Probes). Cells were imaged using a LSM700 Zeiss Confocal microscope at 63,000× magnification.

### Identification of putative sites for S-acylation

Potential S-acylation sites in the syntaxin 11 protein sequence were identified using the CSS-PALM 2.04 software set at a medium threshold [38].

### Acyl-biotinyl exchange assay for S-acylation

S-acylation was assayed using an adaptation of the procedure described by Drisdel and Green (2004) [39]. Briefly, 10^7^ YTS cells were sonicated in lysis buffer [500 mM NaCl, 2.7 mM KCl, 10 mM Na_2_HPO_4_, 1.8 mM KH_2_PO_4_, 5 mM EDTA, 1% (v/v) Triton X-100, complete protease inhibitors] on ice and then centrifuged at 13,000 g in a microcentrifuge. NEM was added to the supernatant to a final concentration of 25 mM and the sample incubated at 4°C for 16 h. Proteins were precipitated from the cell lysate by chloroform methanol precipitation and centrifugation for 10 min at 13,000 g in a microcentrifuge. Protein pellets were resuspended into resuspension buffer [50 mM Tris pH 7.5, 150 mM NaCl, 2% (v/v) sodium dodecyl sulfate, 8 M urea]. To one half of the sample, lysis buffer with 8 mM Biotin-1-Biotinamido-4-[4'-(maleimidomethyl) cyclohexanecarboxamido]butane (Biotin-BMCC, Pierce) was added whilst to the other half lysis buffer with 8 mM Biotin-BMCC and 10% (v/v) hydroxylamine was added. The samples were incubated for 2 hours at 4°C before precipitating and resuspending the proteins as before. The samples were then incubated with neutravidin agarose beads (Pierce) for 2 h at 4°C. Beads were washed with 500 mM NaCl, 2.7 mM KCl, 10 mM Na_2_HPO_4_, 1.8 mM KH_2_PO_4_, 0.1% (v/v) Triton X-100 and bound proteins eluted by incubation in resuspension buffer and by boiling in Laemmli sample buffer.

## Results

### FHL-4 mutations have differential effects on *SNAP23* binding by syntaxin 11, but do not affect Munc18-2 binding

**Figure 1:**
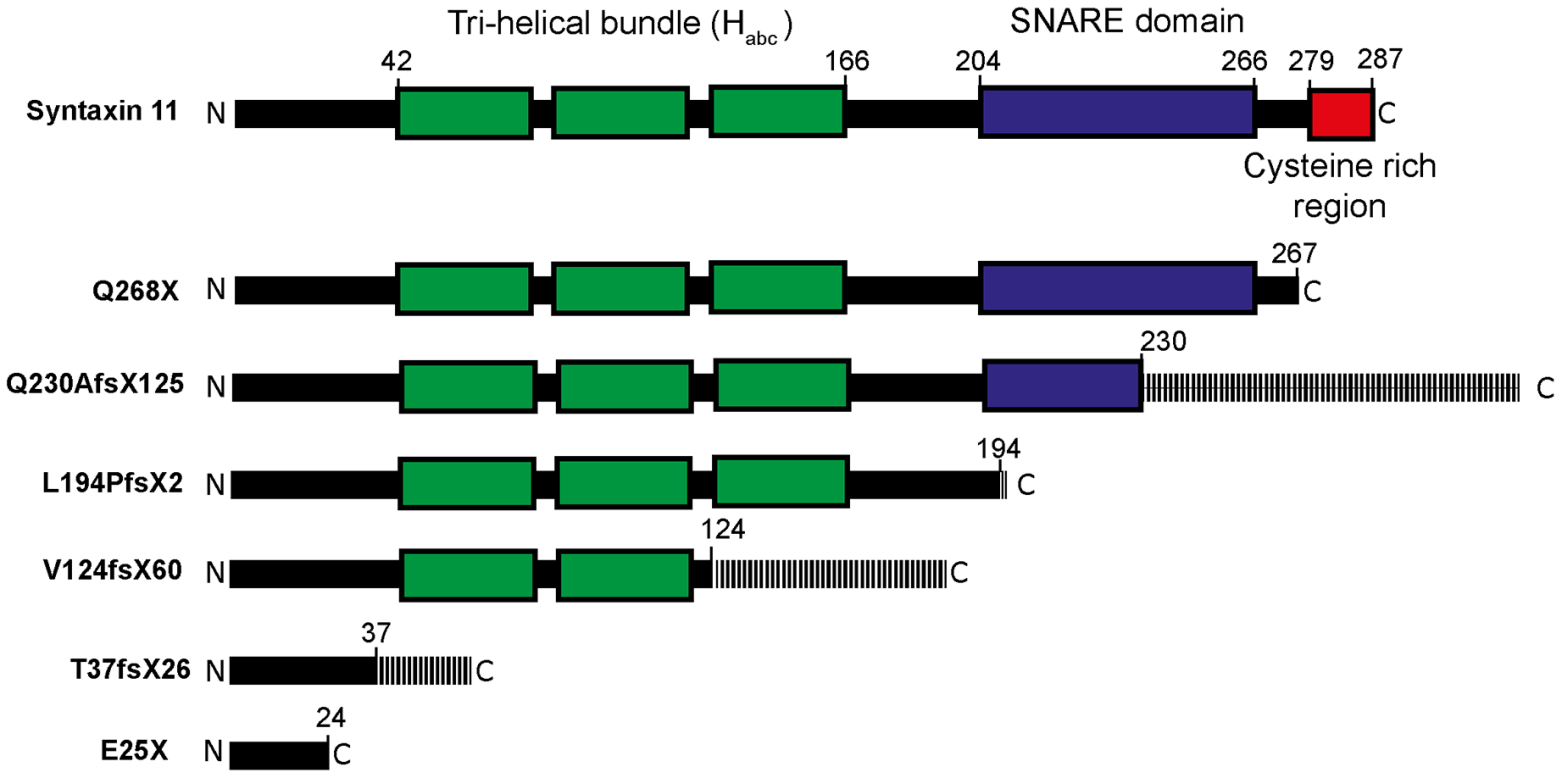
FHL-4 mutations characterised in this study. Schematic of the predicted protein domain structure of syntaxin 11 and FHL-4 mutant proteins. Regions of the FHL-4 mutant proteins encoded as a consequence of frameshift mutations are shown with hatch shading.

**Figure 2:**
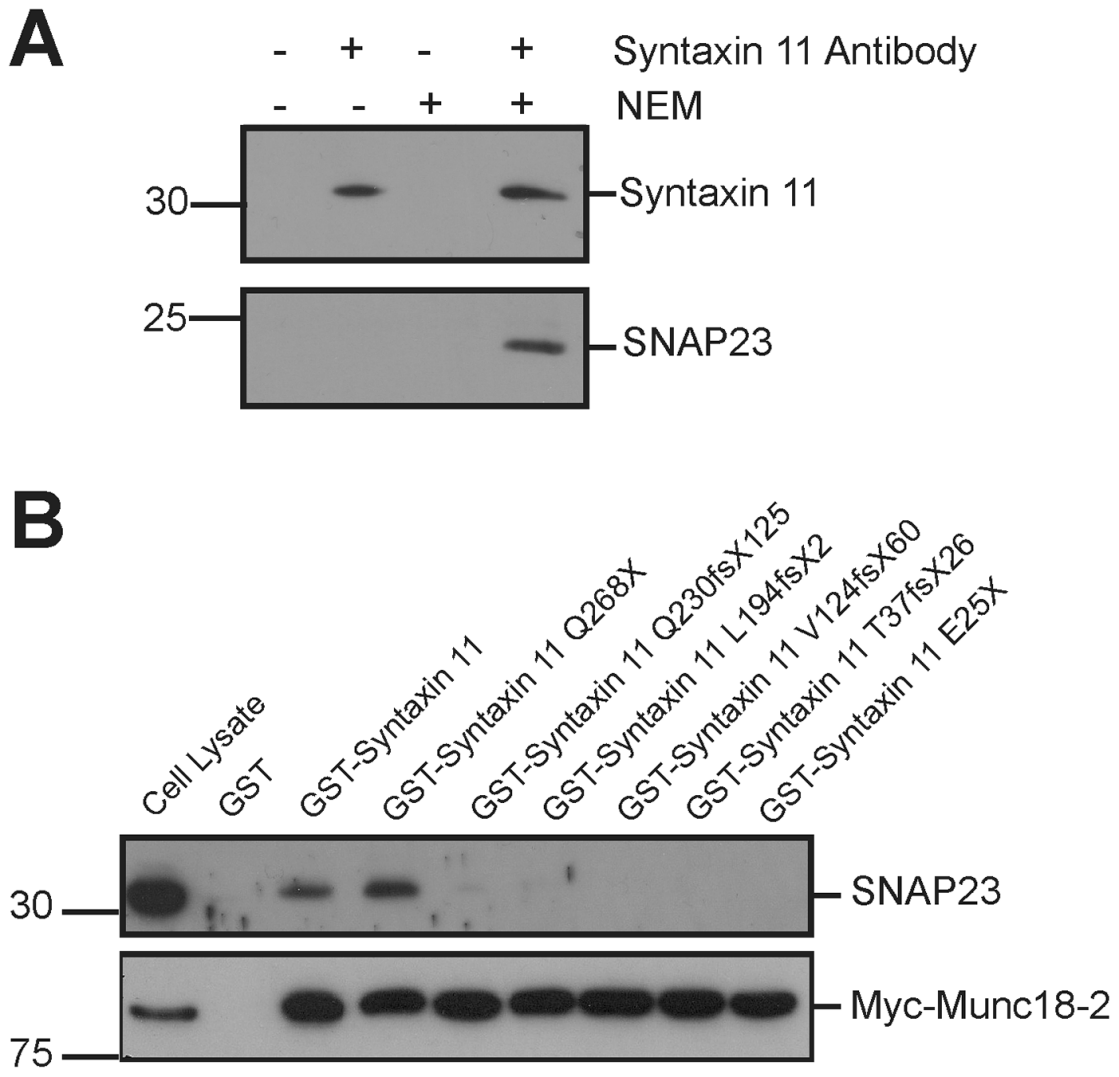
Analysis of the effect of FHL-4 mutations on the interaction of syntaxin 11 with SNAP23 and Munc18-2. (A) Co-immunopreciptation of syntaxin 11 and SNAP23. Prior to lysis, YTS NK cells were pre-incubated in the absence or presence of NEM. Cell lysates were then incubated in the presence or absence of a mouse syntaxin 11 specific antibody and antibody bound proteins pulled down with protein-G sepharose. The precipitated proteins were resolved by SDS-PAGE and immunoblots probed with SNAP23 and rabbit syntaxin 11 specific antibodies. (B) GST pulldowns with syntaxin 11 FHL-4 mutants. GST, GST-syntaxin 11 and GST fusions of FHL-4 mutants were bound to glutathione sepharose beads (See Figure S1 for a coomassie stained gel of the GST fusions bound to the glutathione sepharose beads). Pulldowns of a cell lysate prepared from HeLa-M cells transfected with Myc-Munc18-2 were then performed with GST and the GST-fusions immobilized on glutathione sepharose. The precipitated proteins were resolved by SDS-PAGE and immunoblots probed with SNAP23 and Myc-tag specific antibodies.

The homozygous FHL-4 truncation (Q268X and E25X) and frameshift (Q230fsX125, L194fsX2, V124fsX60 and T37fsX62) mutations (Figure 1) are associated with a defect in secretory lysosome exocytosis and the corresponding loss of NK cytotoxicity [7]–[11], but how these mutations impair the function of syntaxin 11 is poorly understood. We, therefore, studied the effect of these mutations on the interaction of syntaxin 11 with other proteins. Since the SNARE protein SNAP23 binds to syntaxin 11 in other cell types and in platelets [22], [27], [40], we examined whether this interaction also occurs in NK cells. In initial experiments SNAP23 did not co-immunopreciptate with syntaxin 11 from cell lsyates prepared from the model human NK cell line YTS. Therefore, prior to cell lysis, YTS cells were incubated with NEM to inhibit NEM-sensitive factor mediated disassembly of SNARE complexes. Syntaxin 11 antibodies immunoprecipitated syntaxin 11 irrespective of whether the cells were pre-treated with NEM, whereas SNAP23 was only co-immunopreciptated in cell lysates prepared from cells pre-treated with NEM (Figure 2A). These data are consistent with SNAP23 being a binding partner for syntaxin 11 in NK cells. Consequently, SNAP23 was used to determine if SNARE binding was affected by FHL-4 mutations.

The effect of FHL-4 mutations on the interaction of syntaxin 11 with its binding partners were then examined using GST pulldowns of cell lysates prepared from HeLa-M cells transfected with a construct encoding Myc-Munc18-2 (Figure 2B). Whilst a GST fusion of full-length syntaxin 11 bound SNAP23 from cell lysates, no binding was observed for GST alone (Figure 2B). The Q268X truncation mutant protein has an intact SNARE domain (Figure 1) and bound to SNAP23, whereas the Q230fsX125, L194fsX2, V124fsX60, T37fsX62 and E25X mutant proteins, which lack part or all of the SNARE domain (Figure 1) had substantially reduced binding to SNAP23 (Figure 2B). In contrast, all of the syntaxin 11 FHL-4 mutant proteins bound to Myc-Munc18-2 (Figure 2B and S1). This suggests that all of the mutant proteins characterized in this study retain the ability to bind to Munc18-2, due to the presence of a Munc18-2 binding site in the N-terminal 24 residues of syntaxin 11. By contrast, with the exception of Q268X, FHL-4 mutations abrogate binding to SNAP23.

**Figure 3:**
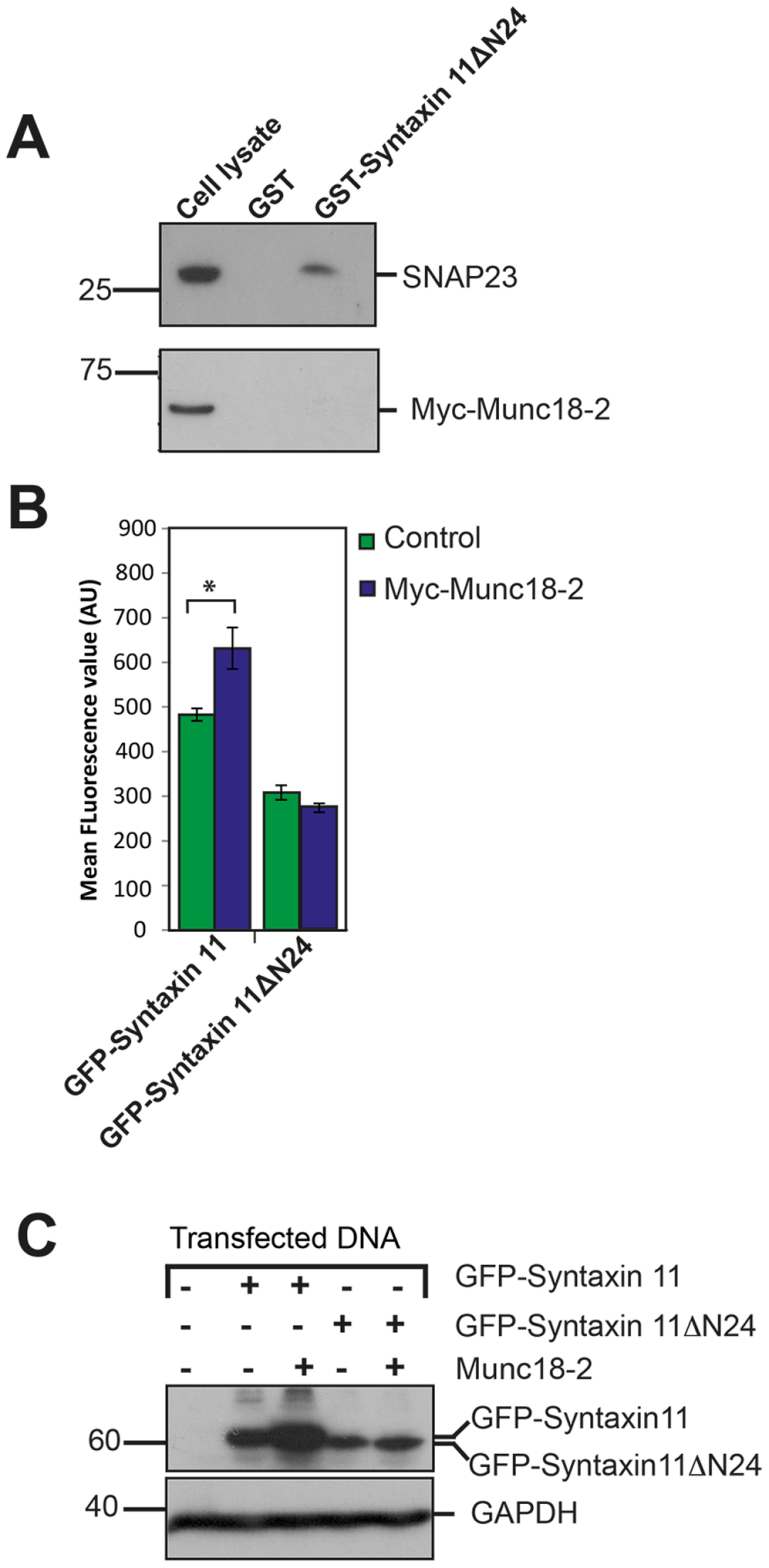
The N-terminal 24 residues of syntaxin 11 are necessary for binding to Munc18-2 and for the stabilisation of syntaxin 11 expression by Munc18-2. (A) GST pulldowns with the syntaxin 11ΔN24 mutant. GST and GST-syntaxin 11ΔN24 were bound to glutathione sepharose (See Figure S2 for a coomassie blue stained gel of the proteins bound to glutathione sepharose). Pulldowns of a cell lysate prepared from HeLa-M cells transfected with Myc-Munc18-2 were then performed with the GST and GST-syntaxin 11ΔN24 immobilized on glutathione sepharose The precipitated proteins were resolved by SDS-PAGE and analysed by immunoblotting with SNAP23 and Myc-tag specific antibodies. (B) Flow cytometric analysis of the effect of Munc18-2 on the expression of syntaxin 11. Constructs encoding GFP-syntaxin 11 or GFP-syntaxin 11ΔN24 were transfected into HeLa-M cells with either a control plasmid construct or a construct encoding Myc-Munc18-2. The mean fluorescence value for the cells was quantified by flow cytometry. Statistical analysis was performed using the Student's t-test. * P = 0.032. Error bars represent standard error of the mean for triplicate samples from 3 independent experiments. AU (arbitrary units). (C) Immunoblot analysis of the effect of Munc18-2 on the expression of syntaxin 11. Cell lysates prepared from the HeLa-M transfectants were analysed by immunoblotting with syntaxin 11, Myc-tag and GAPDH specific antibodies.

Intriguingly, in FHL-5 the level of the syntaxin 11 protein is also reduced [12], [13], consistent with a role for Munc18-2 in stabilizing syntaxin 11 expression. We therefore investigated whether interaction with the N-terminal 24 residues of syntaxin 11 is required for the stabilization of syntaxin 11 expression by Munc18-2. As anticipated a *de novo* mutant of syntaxin 11 that lacked the N-terminal 24 residues (syntaxin 11ΔN24) did not bind to Munc18-2 in a GST pulldown experiment (Figure 3A and S2). We then performed a flow cytometric assay to quantify the effect of Munc18-2 on the expression of syntaxin 11. There was a significant increase in fluorescence of cells when cells transfected with a GFP fusion of syntaxin 11 (GFP-syntaxin 11) were also transfected with Myc-Munc18-2 (Figure 3B). In marked contrast Myc-Munc18-2 did not increase the level of GFP-syntaxin 11ΔN24 (Figure 3B). Immuoblotting confirmed that this increase in cell-associated GFP fluorescence in cells co-expressing Myc-Munc18-2 was due to elevated expression of GFP-syntaxin 11, whereas the level of GFP-syntaxin 11ΔN24 was unaffected by Myc-Munc18-2 (Figure 3C). Thus, the Munc18-2 dependent stabilisation of the expression of syntaxin 11 is dependent on the N-terminal 24 residues of syntaxin 11.

### Membrane association of syntaxin 11 and its polarization to the activating immunological synapse are impaired by the FHL-4 Q268X mutation

**Figure 4:**
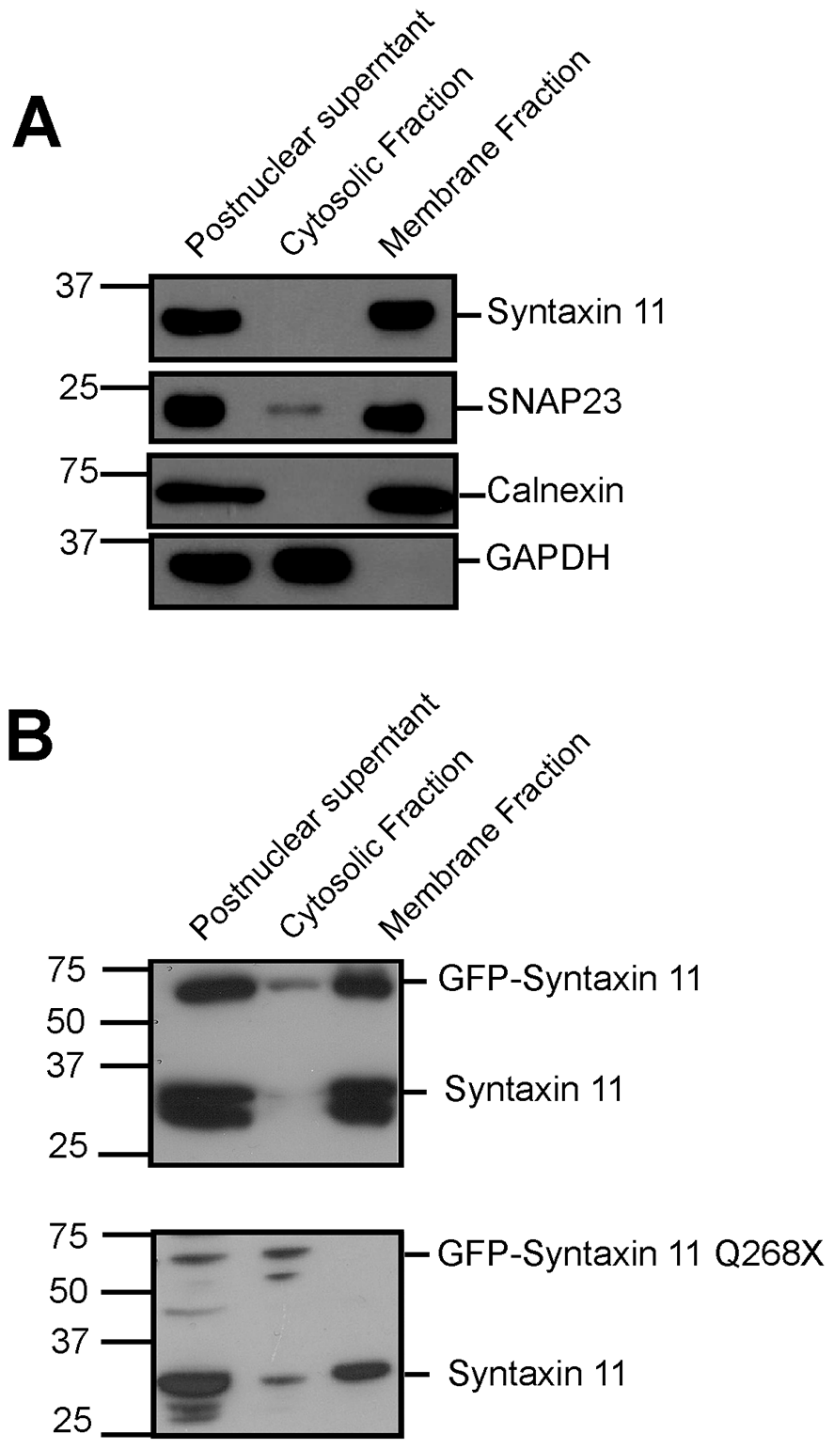
The Q268X mutation abolishes membrane association of syntaxin 11 in YTS NK cells. (A) Analysis of the membrane association of syntaxin 11 expressed endogenously in YTS NK cells. Postnuclear supernatants from YTS NK cells were fractionated by centrifugation into pellet (membrane) and supernatant (cytosolic) fractions. The fractions were resolved by SDS-PAGE and immunoblots probed with antibodies specific for syntaxin 11, SNAP23, calnexin and GAPDH. (B) Analysis of the membrane association of GFP-syntaxin 11 and GFP-syntaxin 11 Q268X in YTS NK cells. Postnuclear supernatants of YTS cells transfected with either GFP-syntaxin 11 or GFP syntaxin 11 Q268X were fractionated by centrifugation into membrane and cytosolic fractions. The fractions were resolved by SDS-PAGE and GFP-syntaxin 11 fusion proteins detected by probing immunoblots with a rabbit syntaxin 11 specific antibody.

The membrane association of SNAREs is critical for their function in membrane fusion reactions [17], [18]. Syntaxin 11 is an atypical SNARE in that it lacks a transmembrane domain, but it has a C-terminal cysteine-rich region that has been suggested as a site for S-acylation (Figure 1) [19]. However, in many cell types this region is not essential for membrane association [19], [22], [41]. We therefore examined the role of the C-terminal cysteine-rich region, which is absent in the FHL-4 mutant protein syntaxin 11 Q268X, in the membrane association and localization of syntaxin 11 in NK cells. Centrifugation of postnuclear supernatants revealed that syntaxin 11, expressed endogenously by the YTS NK cells, fractionated into the membrane enriched fraction, as did the S-acylated protein SNAP23 and the integral membrane protein calnexin, whereas the cytosolic enzyme GAPDH was present in the cytosolic fraction (Figure 4A). Likewise for transfected YTS cells, GFP-syntaxin 11 was present in the membrane fraction (Figure 4B). In contrast the GFP-syntaxin 11 Q268X was in the cytosolic fraction (Figure 4B). The level of GFP-syntaxin 11 Q268X did not exceed that of the endogenous wild type protein, indicating that the presence of the mutant protein in the cytosolic fraction was not due to overexpression (Figure 4B).

**Figure 5:**
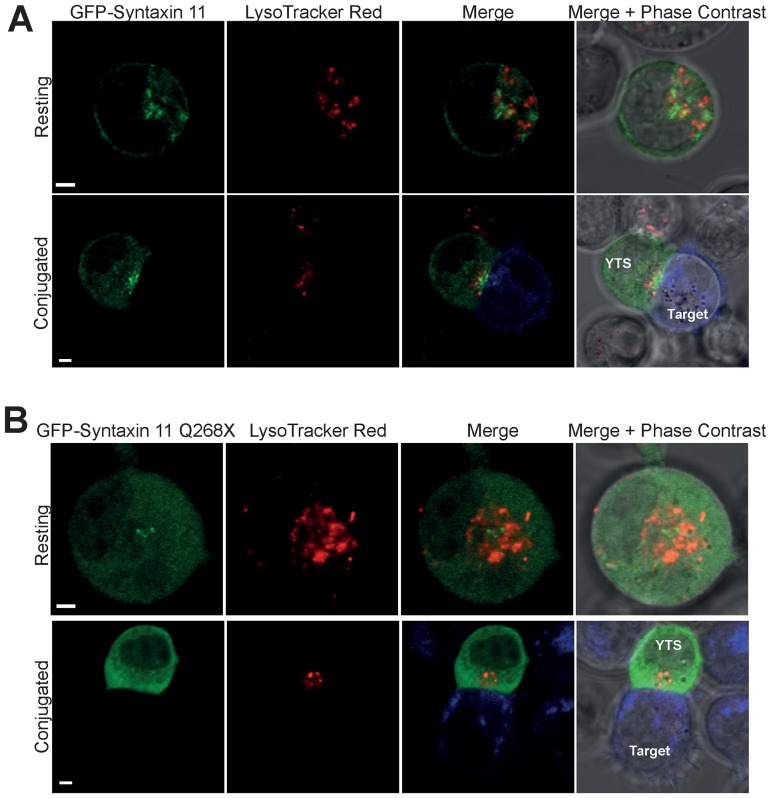
Syntaxin 11 Q268X displays a diffuse cytosolic localisation in resting YTS NK cells and is not recruited to the activating immunological synapse. Analysis of the localization of wild type (A) syntaxin 11 and the Q268X mutant (B) in YTS NK cells. YTS cells transfected with either GFP-syntaxin 11 or GFP-syntaxin 11 Q268X were stained with LysoTracker Red to visualize secretory lysosomes and either imaged immediately (Resting) or conjugated to 721.221 target cells pre-stained with Cell Trace Far Red (blue in the merge image panels). Live cells were imaged using a Zeiss LSM700 laser scanning confocal microscope. Scale bars 5 µm.

The localization of these proteins was also examined by live cell confocal microscopy. Consistent with previous reports [20], [29], GFP-syntaxin 11 was localized predominantly to cytoplasmic punta in resting YTS NK cells that were distinct from acidic compartments labelled with LysoTracker dye (secretory lysosomes) (Figure 5A). In YTS NK cells activated by conjugation to the 721.221 target cells, GFP-syntaxin 11 associated with the cytoplasmic punta was concentrated in the region of the immunological synapse (Figure 5A and S3). In resting YTS NK cells, GFP-syntaxin 11 Q268X displayed a more diffuse cytoplasmic localisation than that of the wild type protein and although in some of the conjugated cells there was an increased concentration of GFP-syntaxin 11 Q268X in proximity to the immunological synapse, a diffuse cytoplasmic distribution of this protein was still evident (Figure 5B and S4). These data demonstrate a critical role for the C-terminal 21 residues in determining the localization of syntaxin 11 in both resting and activated NK cells.

### Wild type syntaxin 11, but not the FHL-4 mutant Q268X, promotes Munc18-2 recruitment to the activating immunological synapse

**Figure 6:**
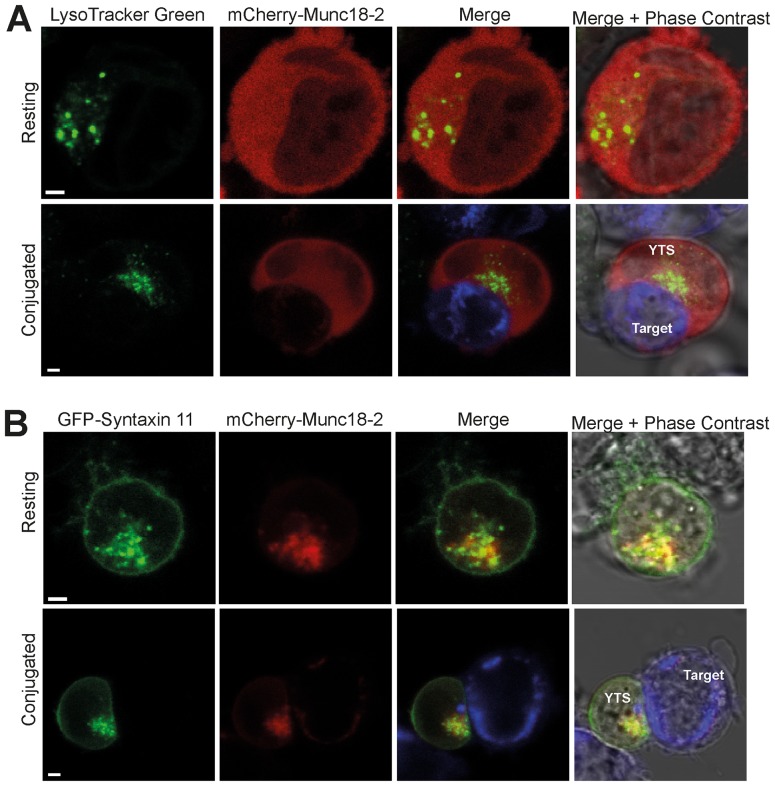
Syntaxin 11 recruits Munc18-2 to cytoplasmic membranes and to the activating immunological synapse. Analysis of the localization of Munc18-2 in YTS NK cells. (A) YTS cells were transfected with mCherry-Munc18-2, stained with LysoTracker Green to visualize secretory lysosomes and either imaged immediately (Resting) or conjugated with 721.221 target cells pre-stained with Cell Trace Far Red (blue in the merge image panels). (B) YTS cells were co-transfected with mCherry-Munc18-2 and GFP-syntaxin 11 and either imaged alone or after incubation with 721.221 cells pre-stained with Cell Trace Far Red. Cells were imaged using a Zeiss LSM700 laser scanning confocal microscope. Scale bars 5 µm.

**Figure 7:**
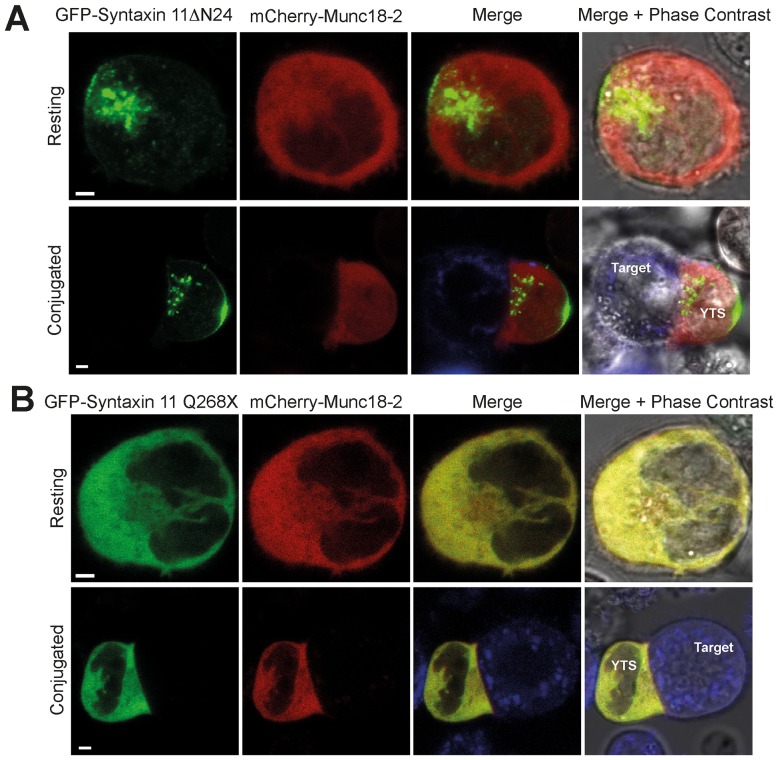
Recruitment of Munc18-2 to cytoplasmic membranes and to the activating immunological synapse is dependent on the N- and C-terminal regions of syntaxin 11. Analysis of the effect of expression of the syntaxin 11ΔN24 and Q268X mutant proteins on the localization of Munc18-2 in YTS NK cells. mCherry-Munc18-2 was co-transfected with either GFP-syntaxin 11ΔN24 (A) or GFP-syntaxin 11 Q268X (B). YTS cells were then imaged in the absence of target cells (Resting) or conjugated to 721.221 target cells pre-stained with Cell Trace Far Red (blue in the merge image panels). Cells were imaged using a Zeiss LSM700 laser scanning confocal microscope. Scale bars 5 µm.

Since syntaxin 11 binds to Munc18-2 and these proteins co-localize in NK cells [12], [13], [23], we examined whether this interaction recruits Munc18-2 to cellular membranes. In the absence of co-transfected GFP-syntaxin 11, a mCherry fusion of Munc18-2 (mCherry-Munc18-2) exhibited a diffuse cytoplasmic localisation in resting YTS NK cells that was unaltered when the YTS cells were activated by conjugation to target cells (Figure 6A and S5). In marked contrast, when co-expressed with GFP-syntaxin 11, both mCherry-Munc18-2 and GFP-syntaxin 11 were localized to cytoplasmic punta in resting YTS NK cells and were polarized to activating immunological synapse in conjugated cells (Figure 6B and S6). Taken together these data suggest that the endogenous syntaxin 11 in YTS NK cells is unable to recruit mCherry-Munc18-2 to cellular membranes, potentially because syntaxin 11 is already bound to the endogenous Munc18-2. Conversely, expression of GFP-syntaxin 11 increases the overall pool of syntaxin 11, enabling mCherry-Munc18-2 to be recruited to membranes. However, although GFP-syntaxin 11ΔN24 was localized to cytoplasmic puncta in resting YTS NK cells and polarized to the activating immunological synapse, co-expressed mCherry-Munc18-2 exhibited a diffuse cytoplasmic localization (Figure 7A and S7). Likewise mCherry-Munc18-2 exhibited a diffuse cytoplasmic localization in cells when co-expressed with GFP-syntaxin 11 Q268X (Figure 7B and S8). Thus, both the N-terminal and C-terminal regions of syntaxin 11 are required for the recruitment Munc18-2 to cytoplasmic membranes and to the activating immunological synapse.

### Syntaxin 11, but not the FHL-4 Q268X mutant, is S-acylated in NK cells

**Figure 8:**
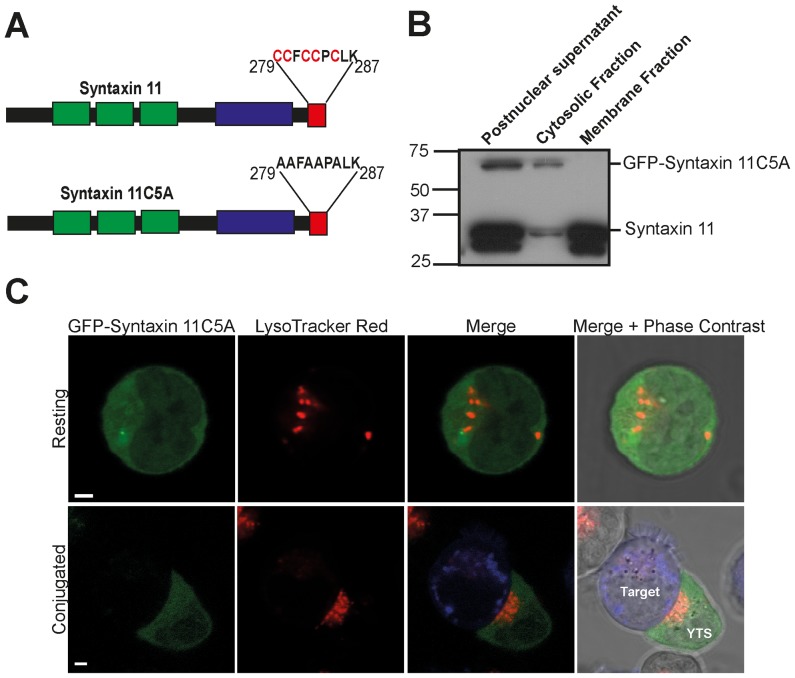
Cysteines in the C-terminal region of syntaxin 11 are required for membrane association. (A) C279, C280, C282, C283 and C285 (shown in red) were predicted by the CSS-PALM 2.04 software [38] to be potential S-acylation sites in syntaxin 11. The corresponding 5 cysteine residues were mutated to alanine to generate the *de novo* mutant syntaxin 11C5A. (B) Analysis of the membrane association of syntaxin 11C5A in YTS NK cells. Postnuclear supernatants from YTS NK cells were fractionated by centrifugation into pellet (membrane) and supernatant (cytosolic) fractions. The fractions were resolved by SDS-PAGE and GFP-syntaxin 11C5A was detected by probing immunoblots with a syntaxin 11 specific antibody. (C) Analysis of the localization of the syntaxin 11C5A mutant in YTS NK cells. YTS cells expressing GFP-syntaxin 11C5A were then imaged in the absence of target cells (Resting) or conjugated to 721.221 cells pre-stained with Cell Trace (blue in the merge image panels). Cells were imaged using a Zeiss LSM700 laser scanning confocal microscope. Scale bars 5 µm.

S-acylation involves the covalent addition of long chain fatty acids to the thiols of cysteine residues and is often termed palmitoylation because the predominant fatty acid used is palmitate [42]. The C-terminal cysteine rich region of syntaxin 11 has been suggested as a site for S-acylation [19]. Correspondingly analysis of the syntaxin 11 protein sequence, using the CSS-PALM software [38], revealed that 5 cysteines within the C-terminal cysteine rich region of syntaxin 11 are potential sites for S-acylation (Figure 8A). Since these cysteines are absent in the syntaxin 11 Q268X mutant protein we examined whether these residues are required for membrane association. The cysteine residues were mutated to alanine (syntaxin 11C5A) (Figure 8A) and the membrane association of this *de novo* mutant protein examined. GFP-syntaxin 11C5A was present in the cytosolic fraction after centrifugation of the postnuclear supernatant (Figure 8B), and displayed a diffuse cytosolic localisation in both resting and conjugated YTS NK cells (Figure 8C and S9), demonstrating that these 5 cysteine residues are required for membrane association.

**Figure 9:**
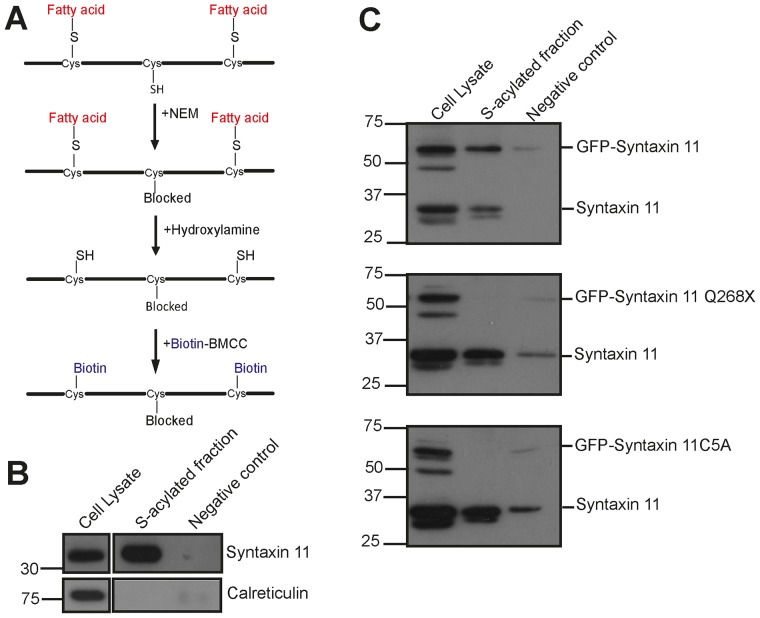
Syntaxin 11 is S-acylated and this is dependent on cysteines in the C-terminal region. (A) Acyl-biotinyl exchange. Free cysteines are blocked with the alkylating agent NEM, long chain fatty acid groups are cleaved from proteins using hydroxylamine revealing free cysteines, which are biotinoylated, enabling proteins to be pulled down with avidin beads. Non-transfected YTS cells (B) and YTS cells transfected with either GFP-syntaxin 11, GFP-syntaxin 11 Q268X or GFP-syntaxin 11C5A (C) were analysed with acyl-biotinyl exchange. Precipitated proteins from samples that had been incubated in the absence (negative control) or presence of hydroxylamine (S-acylated fraction) were resolved by SDS-PAGE. Syntaxin 11 and GFP-syntaxin 11 fusions were detected by probing immunoblots with syntaxin 11 specific antibodies. The non-S-acylated protein calreticulin served as a negative control and was detected with a rabbit anti-calreticulin antibody.

To confirm that syntaxin 11 is S-acylated in NK cells acyl-biotinyl exchange was used [39]. In this procedure hydroxylamine cleaves long chain fatty acids from proteins to reveal free cysteines that can be biotinylated, enabling these proteins to be pulled down using avidin beads (Figure 9A). Syntaxin 11 expressed endogenously by YTS NK cells was pulled down from cell lysates treated with hydroxylamine (Figure 9B), demonstrating that syntaxin 11 is S-acylated in YTS NK cells (Figure 9B). GFP-syntaxin 11 was also pulled down from hydroxylamine treated cell lysates prepared from transfected YTS cells (Figure 9C). In contrast, neither GFP-syntaxin 11C5A nor GFP-syntaxin 11 Q268X were pulled down (Figure 9C). These data demonstrate that cysteine residues in the C-terminal region of the protein, absent in all of the FHL-4 mutants characterised herein, are required for S-acylation of syntaxin 11. Thus, S-acylation is required for the membrane association of syntaxin 11 in NK cells and FHL-4 mutations that delete the C-terminal cysteine rich region correspondingly abolish this posttranslational modification.

## Discussion

**Figure 10:**
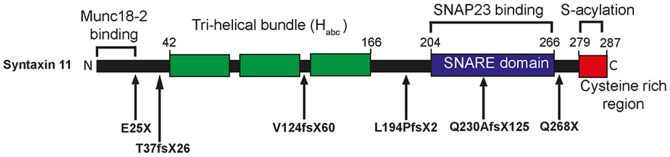
FHL-4 mutations highlight important functional regions of syntaxin 11. The N-terminal 24 residues of syntaxin 11 are required for binding to Munc18-2, the stabilization of syntaxin 11 expression by Munc18-2 and for the membrane recruitment of Munc18-2. The SNARE domain of syntaxin 11 is required for the interaction with the SNARE SNAP23. Cysteine residues within the C-terminal region are required for the S-acylation, membrane association and polarisation of syntaxin 11 to the activating immunological synapse in activated NK cells. The sites of each the FHL-4 mutations studied are indicated.

In this study we used disease-associated mutations to dissect the sequence requirements for the interaction of syntaxin 11 with SNAP23, Munc18-2 and cellular membranes (Figure 10) and hence determine how FHL-4 frameshift and truncation mutations result in the loss of function of syntaxin 11 in NK cells.

We show that SNAP23 is a binding partner of syntaxin 11 in NK cells. This interaction has also been reported in B cells, as well as for syntaxin 11 transfected into J774 macrophages and HeLa cells [22]. By contrast, in RAW 264.7 macrophages transfected with syntaxin 11, SNAP23 binding was not observed and instead syntaxin 11 forms a non-classical SNARE complex that sequesters Vti1b [41]. The SNARE binding partners, and functions, of syntaxin 11 may therefore be cell type specific. Notably, syntaxin 11 also binds to SNAP23 in platelets, in which both proteins are required for granule secretion [27]. FHL-4 mutant proteins, in which there is a partial or complete deletion of the SNARE domain, had reduced binding to SNAP23. Conversely the syntaxin 11 Q268X mutant protein retains an intact SNARE domain and was able to bind to SNAP23 in GST pulldowns. This suggests, that in many cases of FHL-4, reduced binding to SNAP23 may be a factor in the inability of secretory lysosomes to fuse with the plasma membrane in NK cells.

Syntaxin 11 also binds Munc18-2, an interaction that is dependent on an acidic region and the hydrophobic pocket of the SM protein [12], [13], [23], [30]. Whereas the recently identified FHL-4 mutant protein syntaxin 11 L58P is unable to bind to Munc18-2 [16], GST-fusions of the FHL-4 mutant proteins characterised herein, bound to Munc18-2. These data are consistent with the retention of an accessible Munc18-2 binding site by FHL-4 frameshift and truncation mutant proteins. This region in syntaxins is known as the N-peptide and is a key site for interaction with cognate SM proteins [43]–[48]. During the preparation of this manuscript the crystal structure of Munc18-2 was reported and consistent with our data the N-peptide of syntaxin 11 was shown to be critically important for the interaction between this SNARE and Munc18-2 [30].

SM protein binding has multiple functions, including the chaperoning of syntaxins [31]–[36]. Indeed, the chaperone function for Munc18-2 is highlighted in FHL-5, in which mutations in Munc18-2 result in reduced levels of syntaxin 11 [12], [13]. Our data demonstrate that the N-terminal 24 residues of syntaxin 11 are required for the stabilization of the expression of this SNARE by Munc18-2. In addition to stabilizing the expression of cognate SNAREs, the chaperone function of SM proteins can also facilitate the trafficking of SNAREs to specific intracellular compartments [43]–[48]. Indeed, Munc18-2 expression redistributes syntaxin 3 to the plasma membrane in syntaxin 11 deficient murine CTLs [30]. However, we observed that the deletion of the N-terminal region of syntaxin 11 prevented the recruitment of Munc18-2, but not of syntaxin 11, to intracellular membranes in resting NK cells and to the immunological synapse in conjugated NK cells. Furthermore, Munc18-2 exhibited a diffuse cytoplasmic distribution in NK cells that co-express the cytosolic syntaxin 11 Q268X mutant. Thus, our data suggest that syntaxin 11 determines the intracellular localization of Munc18-2 in NK cells.

Despite having no transmembrane domain, syntaxin 11 is membrane associated in NK cells, which we show is dependent on the cysteine-rich C-terminal region. This implies that FHL-4 truncation and frameshift mutant proteins are non-functional in NK cells, at least in part, because they cannot associate with membranes, a prerequisite for SNARE proteins to promote membrane fusion reactions [17],[18]. The C-terminal cysteine rich region is not required for membrane association in all cell types, as deletion of this region does not prevent membrane association in macrophages, NRK cells and HeLa cells [19], [22], [41]. Membrane association in the absence of the C-terminal region in other cell types may be due to interactions with membrane proteins, such as other SNAREs, and may reflect cell-type specific behaviour not observed in NK cells. In addition, we show that syntaxin 11 is S-acylated in NK cells and that this is dependent on cysteine residues in the C-terminal region of the protein. Based on these observations we predict that S-acylation will not only be abolished in the Q268X mutant protein, but in all other FHL-4 truncation and frameshift mutants of syntaxin 11. A number of other SNAREs are S-acylated [49]–[52], both SNAP23 and its neuronal homologue SNAP25, which lack transmembrane domains, use this posttranslational modification for membrane association [49]. Intriguingly, syntaxins 1a, 1b, 6, 7, 8 and 12, all of which have transmembrane domains, are S-acylated [50]–[52]. Removal of the S-acylation site reduced the cycling of syntaxin 7 between the plasma membrane and endosomes [50]. Thus, S-acylation of syntaxin 11 may not only be important for its interaction with membranes, but also for the trafficking of the protein in the cell. Indeed, deletion or mutation of the cysteine rich domain prevents polarization of syntaxin 11 to the immunological synapse, although this is most likely secondary to the inability of these mutant proteins to associate with membranes.

In summary the loss of syntaxin 11 function in FHL-4 frameshift and deletion mutants is associated with the inability of the mutant proteins to associate with membranes, precluding not only the polarization of syntaxin 11 to the activating immunological synapse, but also that of Munc18-2.

## Supporting Information

Figure S1 **Coomassie blue stained gel of GST fusions of wild type syntaxin 11 and FHL-4 mutant proteins bound to glutathione sepharose beads.** GST, GST-syntaxin 11 or GST fusions of FHL-4 mutants were bound to glutathione sepharose. Bound proteins were eluted, resolved by SDS-PAGE and stained with coomassie blue.
(TIF)

Figure S2 **Coomassie blue stained gel of GST fusions of syntaxin 11ΔN24 mutant bound to glutathione sepharose beads.** GST or GST-syntaxin 11ΔN24 were bound to glutathione sepharose. Bound proteins were eluted, resolved by SDS-PAGE and stained with coomassie blue.
(TIF)

Figure S3 **Additional images of the localization of GFP-syntaxin 11 in resting and conjugated YTS NK cells.** YTS cells transfected with GFP-syntaxin 11 were stained with LysoTracker Red to visualize secretory lysosomes and either imaged immediately (resting) or conjugated to 721.221 target cells pre-stained with Cell Trace Far Red (blue in the merge image panels). Live cells were imaged using a Zeiss LSM700 laser scanning confocal microscope. Scale bars 5 µm.
(TIF)

Figure S4 **Additional images of the localization of GFP-syntaxin 11 Q268X in resting and conjugated YTS NK cells.** YTS cells transfected with GFP-syntaxin 11 Q268X were stained with LysoTracker Red to visualize secretory lysosomes and either imaged immediately (Resting) or conjugated to 721.221 target cells pre-stained with Cell Trace Far Red (blue in the merge image panels). Live cells were imaged using a Zeiss LSM700 laser scanning confocal microscope. Scale bars 5 µm.
(TIF)

Figure S5 **Additional images of the localization of mCherry-Munc18-2 in YTS NK cells.** YTS cells were transfected with mCherry-Munc18-2, stained with LysoTracker Green to visualize secretory lysosomes and either imaged immediately (Resting) or conjugated with 721.221 target cells pre-stained with Cell Trace Far Red (blue in the merge image panels). Cells were imaged using a Zeiss LSM700 laser scanning confocal microscope. Scale bars 5 µm.
(TIF)

Figure S6 **Additional images of the localization of mCherry-Munc18-2 in YTS NK cells co-transfected with GFP-syntaxin 11.** YTS cells were co-transfected with mCherry-Munc18-2 and GFP-syntaxin 11 and either imaged alone (Resting) or after incubation with 721.221 cells pre-stained with Cell Trace Far Red. Cells were imaged using a Zeiss LSM700 laser scanning confocal microscope. Scale bars 5 µm.
(TIF)

Figure S7 **Additional images of the localization of mCherry-Munc18-2 in YTS NK cells co-transfected with either GFP-syntaxin 11ΔN24.** mCherry-Munc18-2 was co-transfected with GFP-syntaxin 11ΔN24. YTS cells were then imaged in the absence of target cells (Resting) or conjugated to 721.221 target cells pre-stained with Cell Trace Far Red (blue in the merge image panels). Cells were imaged using a Zeiss LSM700 laser scanning confocal microscope. Scale bars 5 µm.
(TIF)

Figure S8 **Additional images of the localization of mCherry-Munc18-2 in YTS NK cells co-transfected with GFP-syntaxin 11 Q268X.** mCherry-Munc18-2 was co-transfected with GFP-syntaxin 11 Q268X. YTS cells were then imaged in the absence of target cells (Resting) or conjugated to 721.221 target cells pre-stained with Cell Trace Far Red (blue in the merge image panels). Cells were imaged using a Zeiss LSM700 laser scanning confocal microscope. Scale bars 5 µ µm.
(TIF)

Figure S9 **Additional images of the localization of GFP-syntaxin 11 C5A in YTS NK cells.** YTS cells expressing GFP-syntaxin 11C5A were then imaged in the absence of target cells (resting) or conjugated to 721.221 cells pre-stained with Cell Trace (blue in the merge image panels). Cells were imaged using a Zeiss LSM700 laser scanning confocal microscope. Scale bars 5 µm.
(TIF)
