# Supplementary figures and images for: Analysis of Familial Hemophagocytic Lymphohistiocytosis Type 4 (FHL-4) Mutant Proteins Reveals that S-Acylation Is Required for the Function of Syntaxin 11 in Natural Killer Cells
Source: PLoS One. 2014 Jun 9;9(6):e98900. doi: 10.1371/journal.pone.0098900 (PMC4049605; doi:10.1371/journal.pone.0098900)

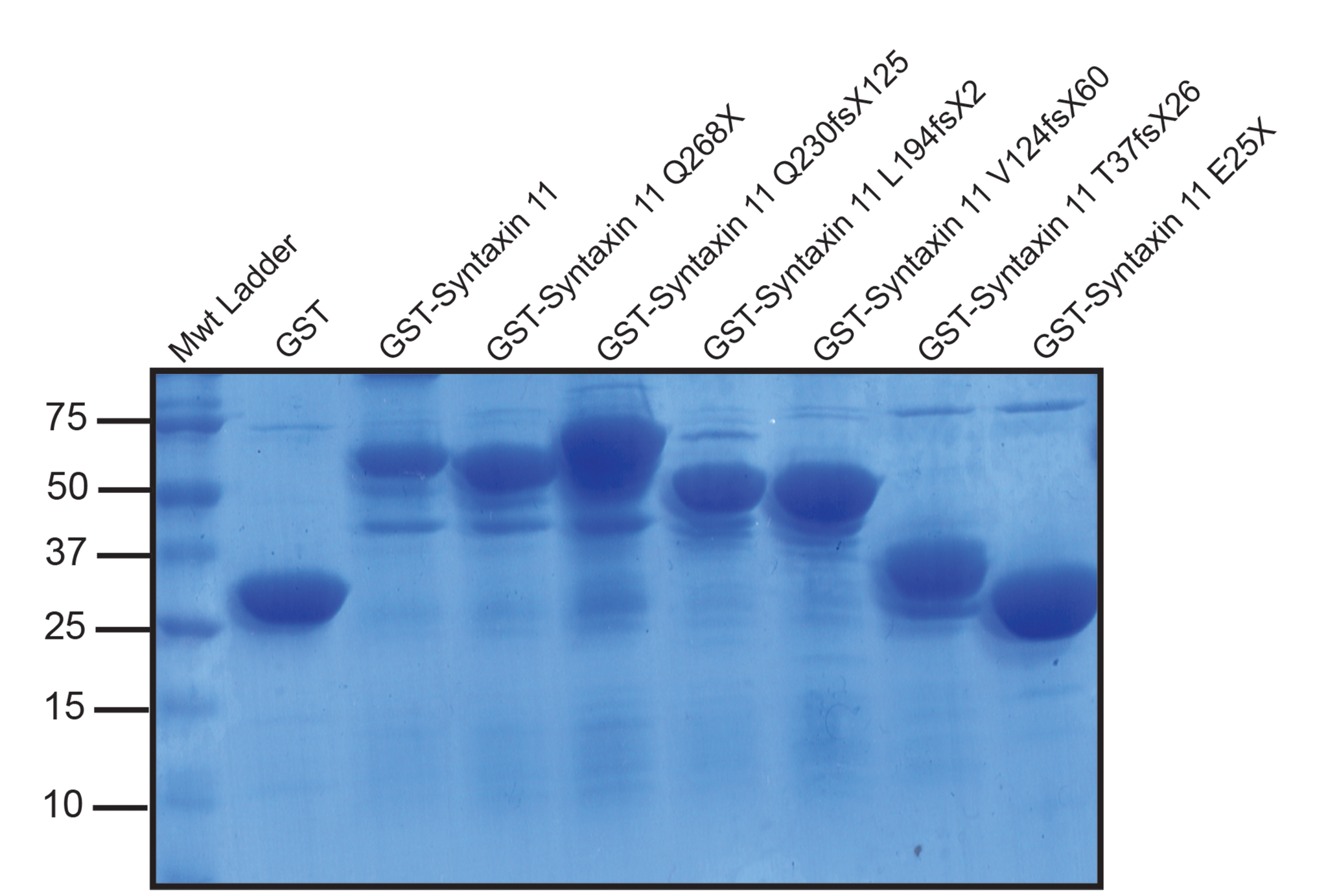

Supplement: Figure S1 — Coomassie blue stained gel of GST fusions of wild type syntaxin 11 and FHL-4 mutant proteins bound to glutathione sepharose beads. GST, GST-syntaxin 11 or GST fusions of FHL-4 mutants were bound to glutathione sepharose. Bound proteins were eluted, resolved by SDS-PAGE and stained with coomassie blue. (TIF) [file pone.0098900.s001.tif]

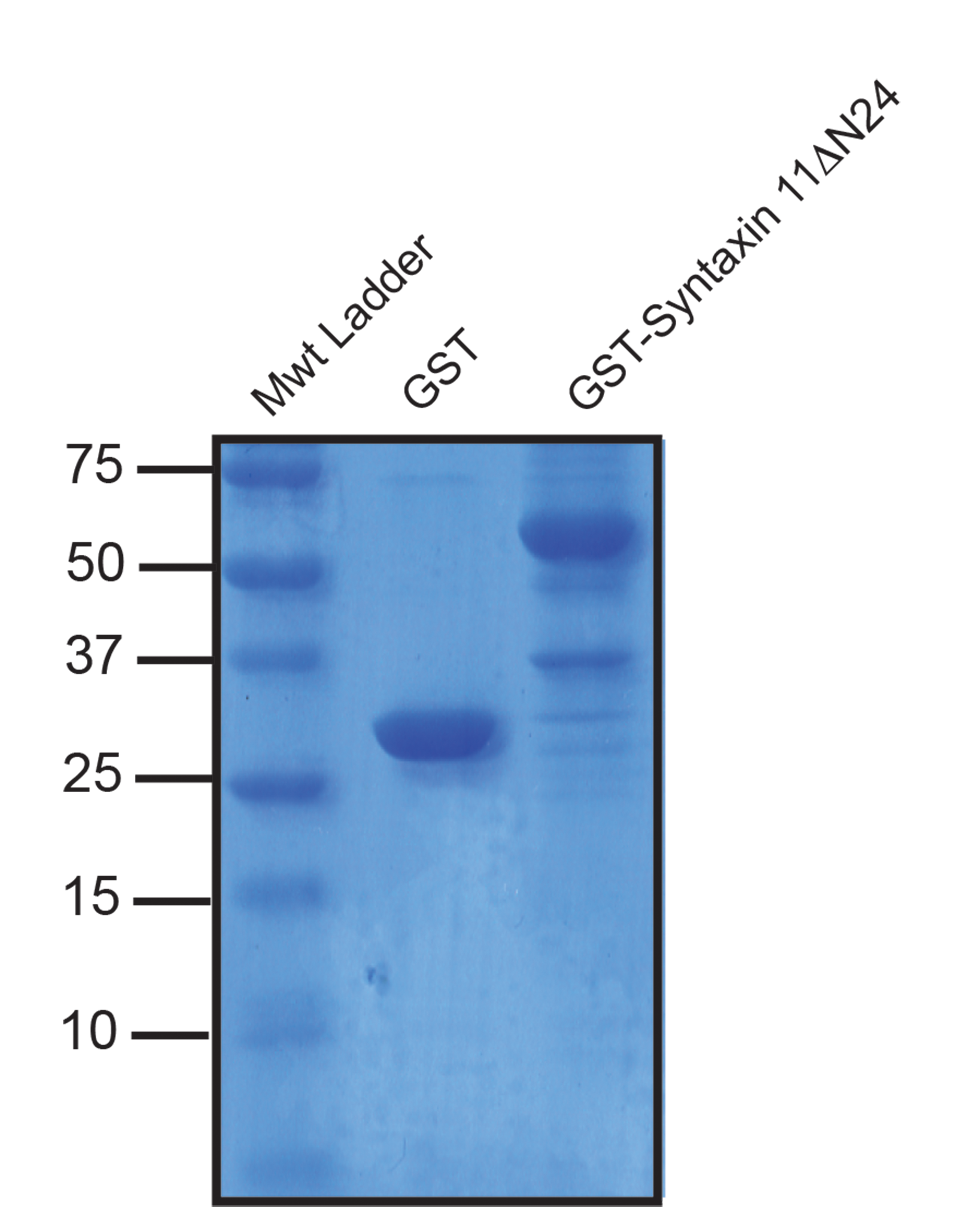

Supplement: Figure S2 — Coomassie blue stained gel of GST fusions of syntaxin 11ΔN24 mutant bound to glutathione sepharose beads. GST or GST-syntaxin 11ΔN24 were bound to glutathione sepharose. Bound proteins were eluted, resolved by SDS-PAGE and stained with coomassie blue. (TIF) [file pone.0098900.s002.tif]

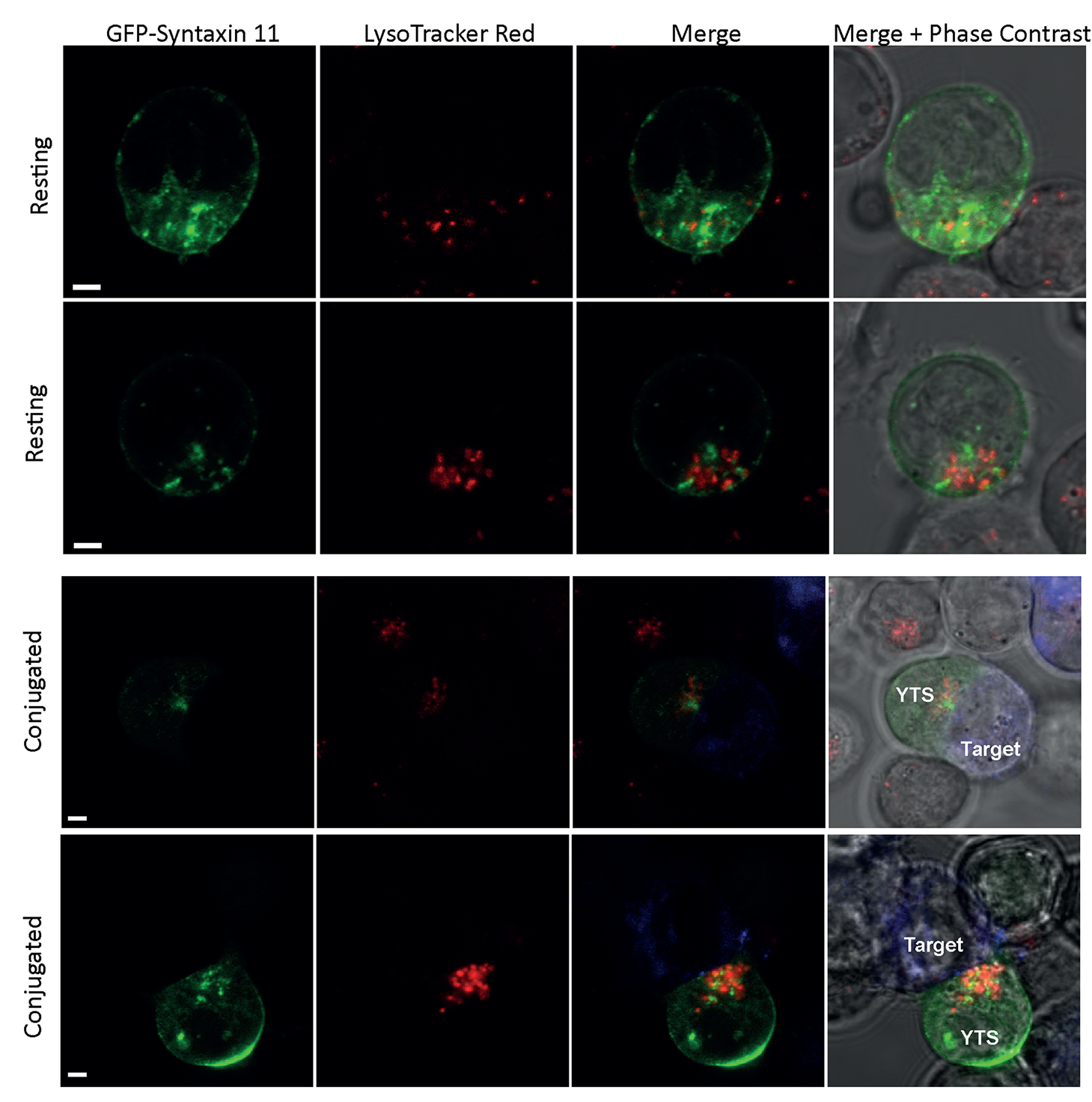

Supplement: Figure S3 — Additional images of the localization of GFP-syntaxin 11 in resting and conjugated YTS NK cells. YTS cells transfected with GFP-syntaxin 11 were stained with LysoTracker Red to visualize secretory lysosomes and either imaged immediately (resting) or conjugated to 721.221 target cells pre-stained with Cell Trace Far Red (blue in the merge image panels). Live cells were imaged using a Zeiss LSM700 laser scanning confocal microscope. Scale bars 5 µm. (TIF) [file pone.0098900.s003.tif]

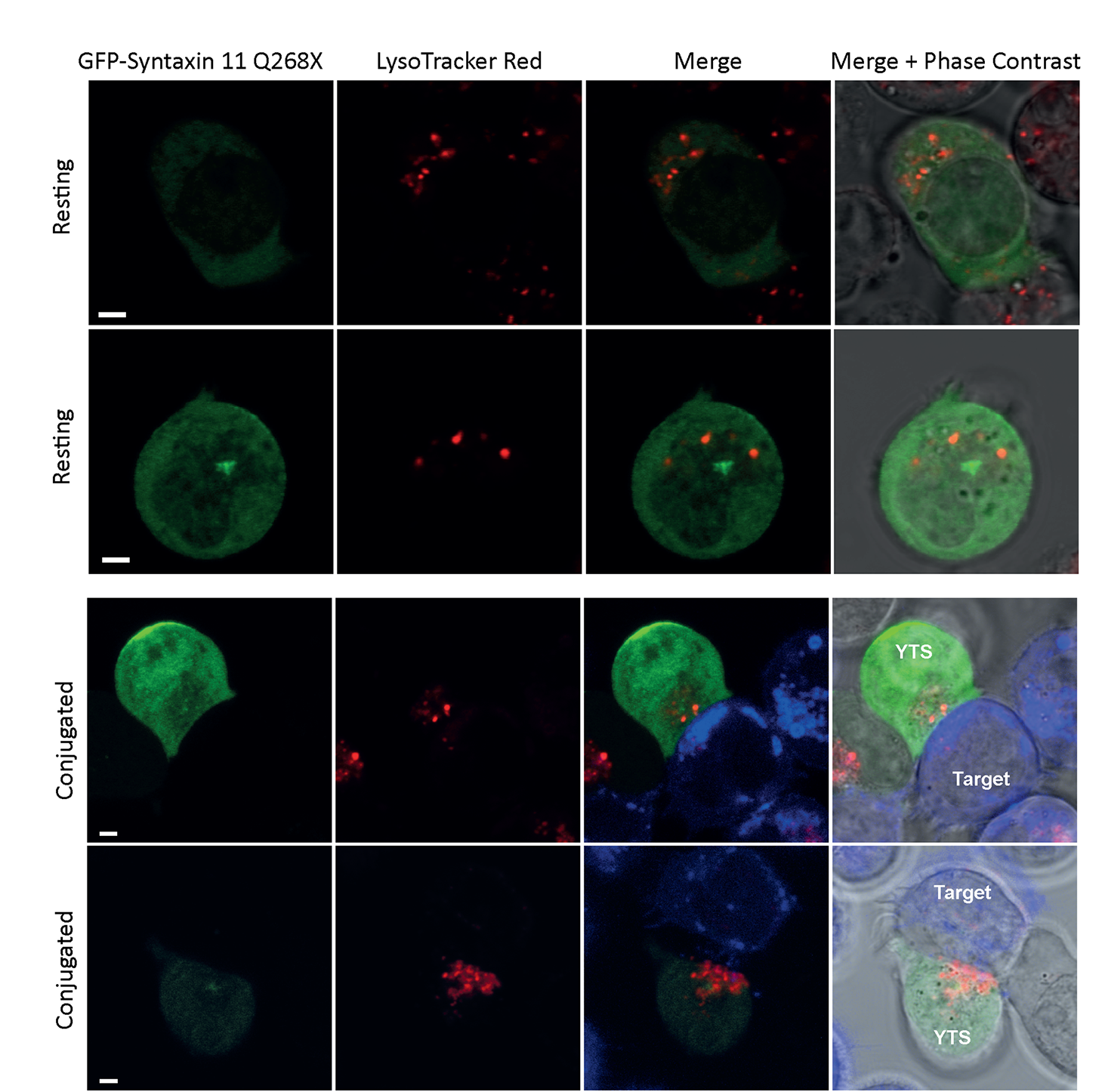

Supplement: Figure S4 — Additional images of the localization of GFP-syntaxin 11 Q268X in resting and conjugated YTS NK cells. YTS cells transfected with GFP-syntaxin 11 Q268X were stained with LysoTracker Red to visualize secretory lysosomes and either imaged immediately (Resting) or conjugated to 721.221 target cells pre-stained with Cell Trace Far Red (blue in the merge image panels). Live cells were imaged using a Zeiss LSM700 laser scanning confocal microscope. Scale bars 5 µm. (TIF) [file pone.0098900.s004.tif]

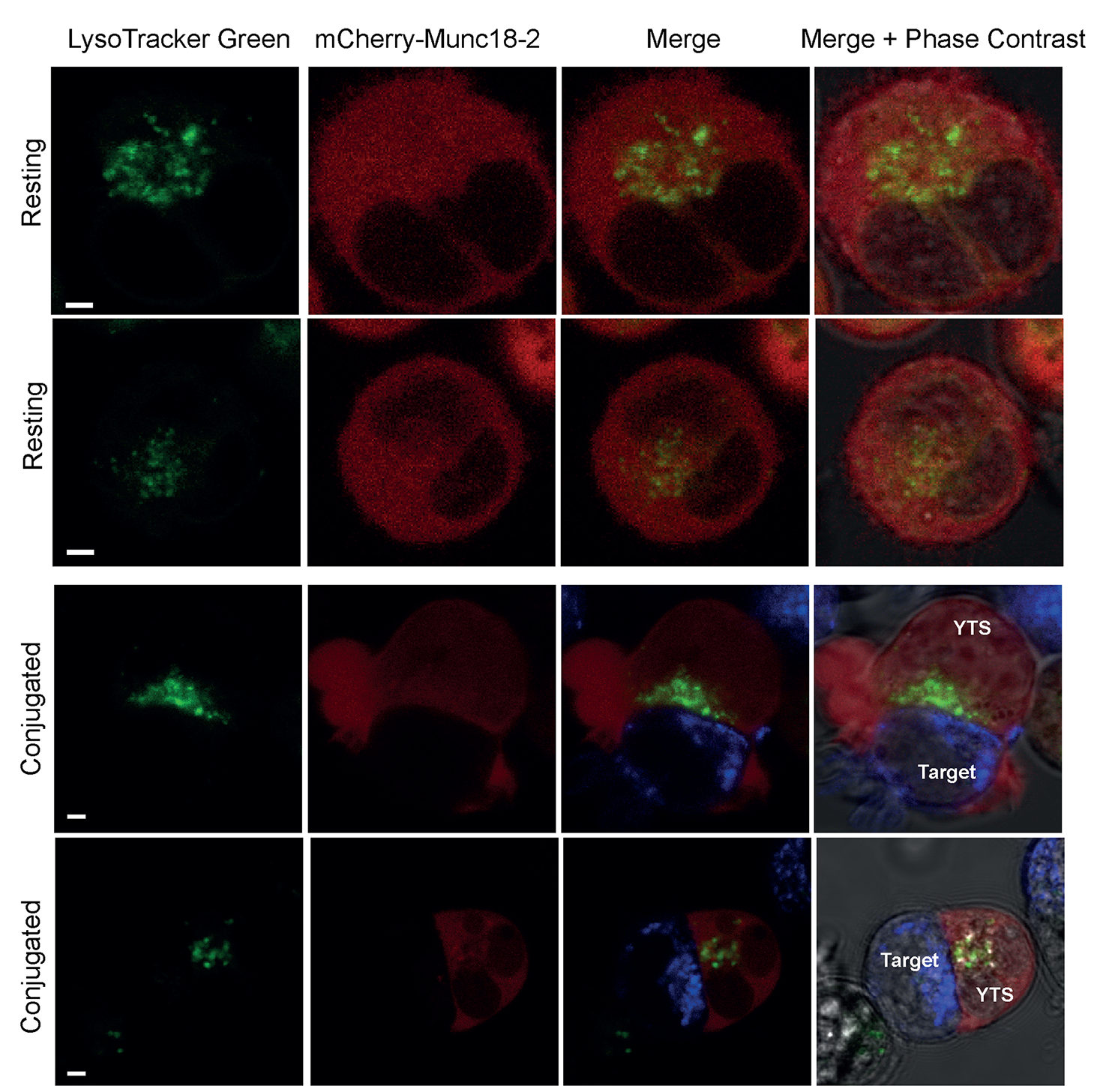

Supplement: Figure S5 — Additional images of the localization of mCherry-Munc18-2 in YTS NK cells. YTS cells were transfected with mCherry-Munc18-2, stained with LysoTracker Green to visualize secretory lysosomes and either imaged immediately (Resting) or conjugated with 721.221 target cells pre-stained with Cell Trace Far Red (blue in the merge image panels). Cells were imaged using a Zeiss LSM700 laser scanning confocal microscope. Scale bars 5 µm. (TIF) [file pone.0098900.s005.tif]

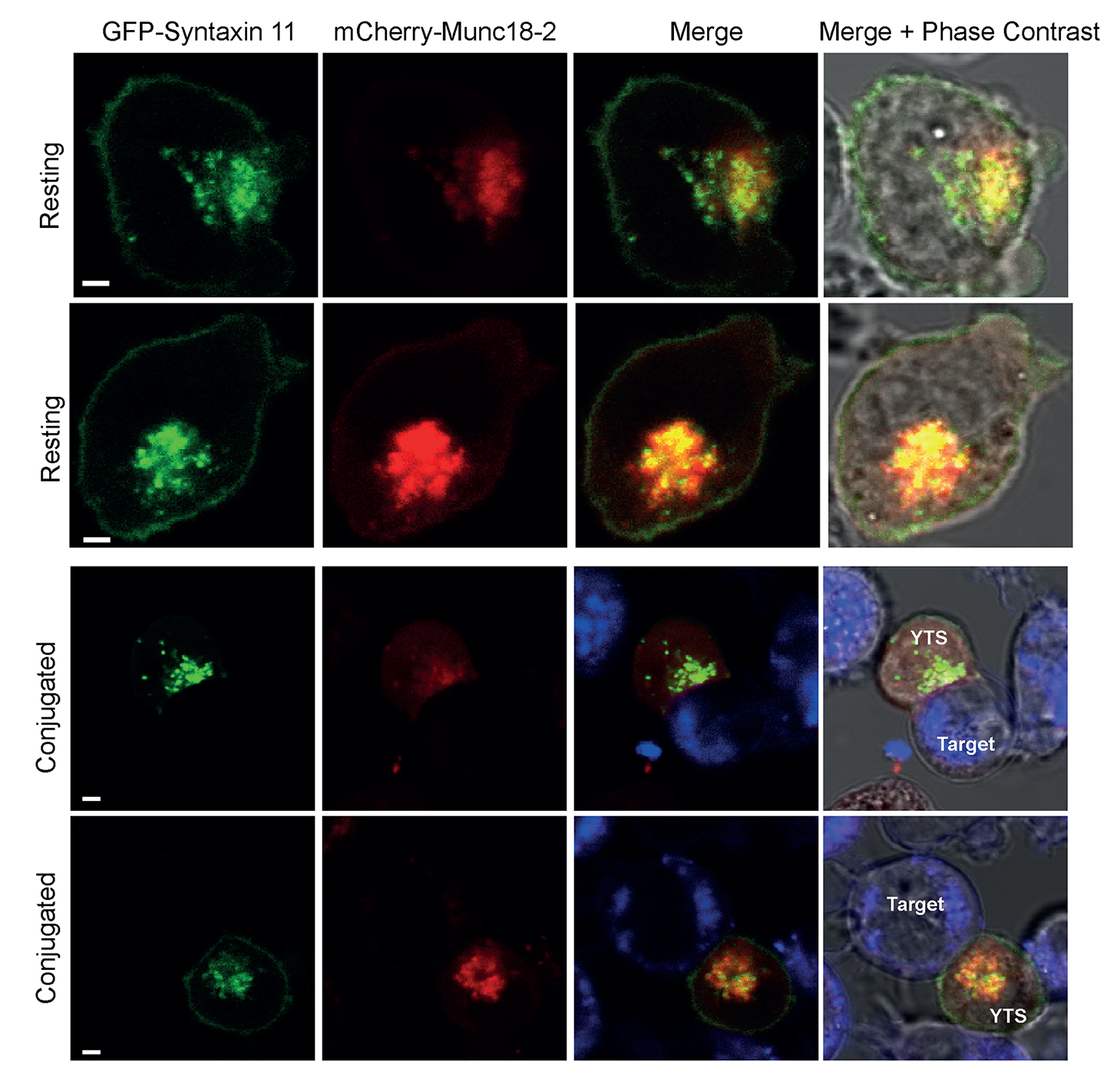

Supplement: Figure S6 — Additional images of the localization of mCherry-Munc18-2 in YTS NK cells co-transfected with GFP-syntaxin 11. YTS cells were co-transfected with mCherry-Munc18-2 and GFP-syntaxin 11 and either imaged alone (Resting) or after incubation with 721.221 cells pre-stained with Cell Trace Far Red. Cells were imaged using a Zeiss LSM700 laser scanning confocal microscope. Scale bars 5 µm. (TIF) [file pone.0098900.s006.tif]

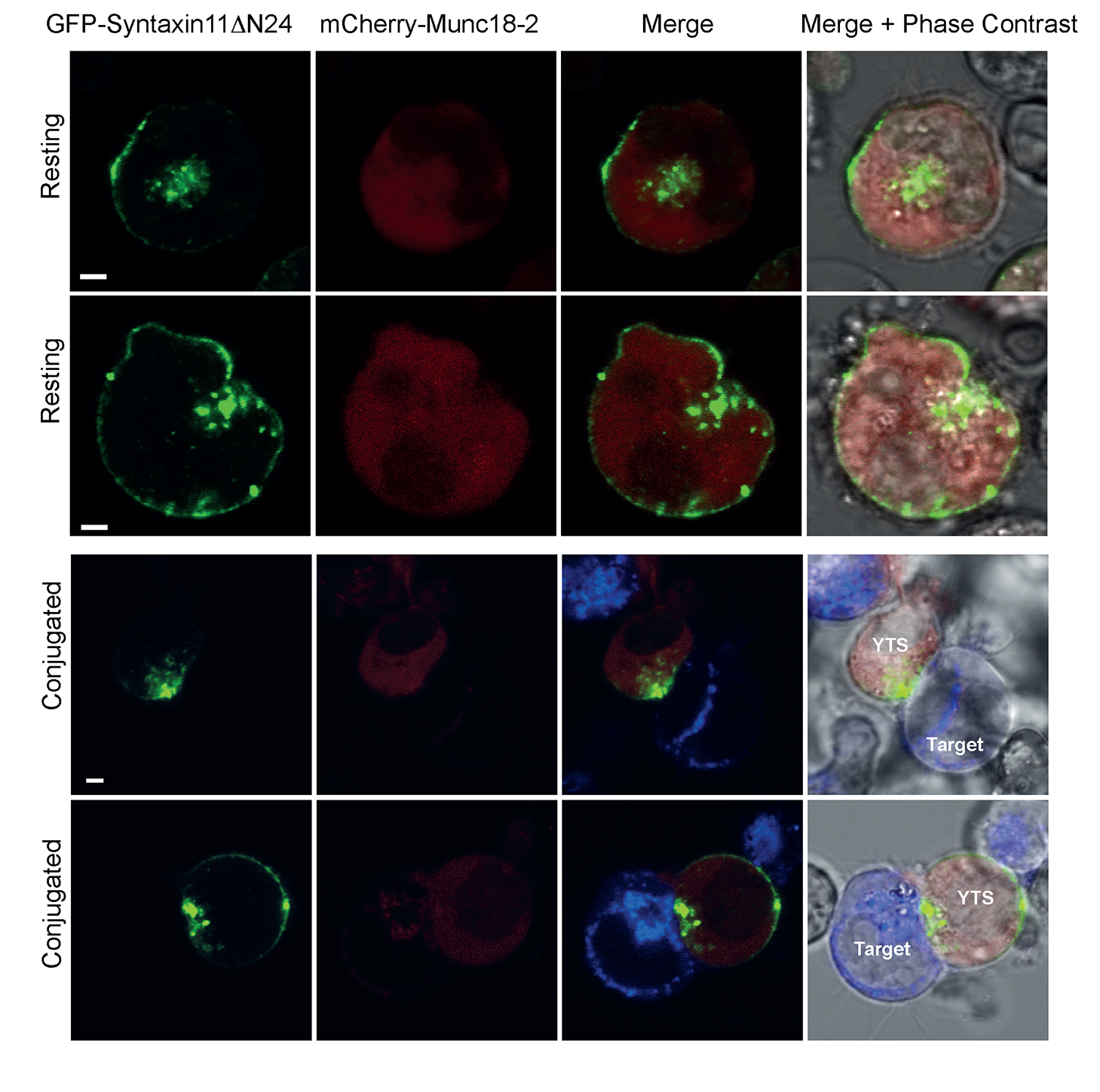

Supplement: Figure S7 — Additional images of the localization of mCherry-Munc18-2 in YTS NK cells co-transfected with either GFP-syntaxin 11ΔN24. mCherry-Munc18-2 was co-transfected with GFP-syntaxin 11ΔN24. YTS cells were then imaged in the absence of target cells (Resting) or conjugated to 721.221 target cells pre-stained with Cell Trace Far Red (blue in the merge image panels). Cells were imaged using a Zeiss LSM700 laser scanning confocal microscope. Scale bars 5 µm. (TIF) [file pone.0098900.s007.tif]

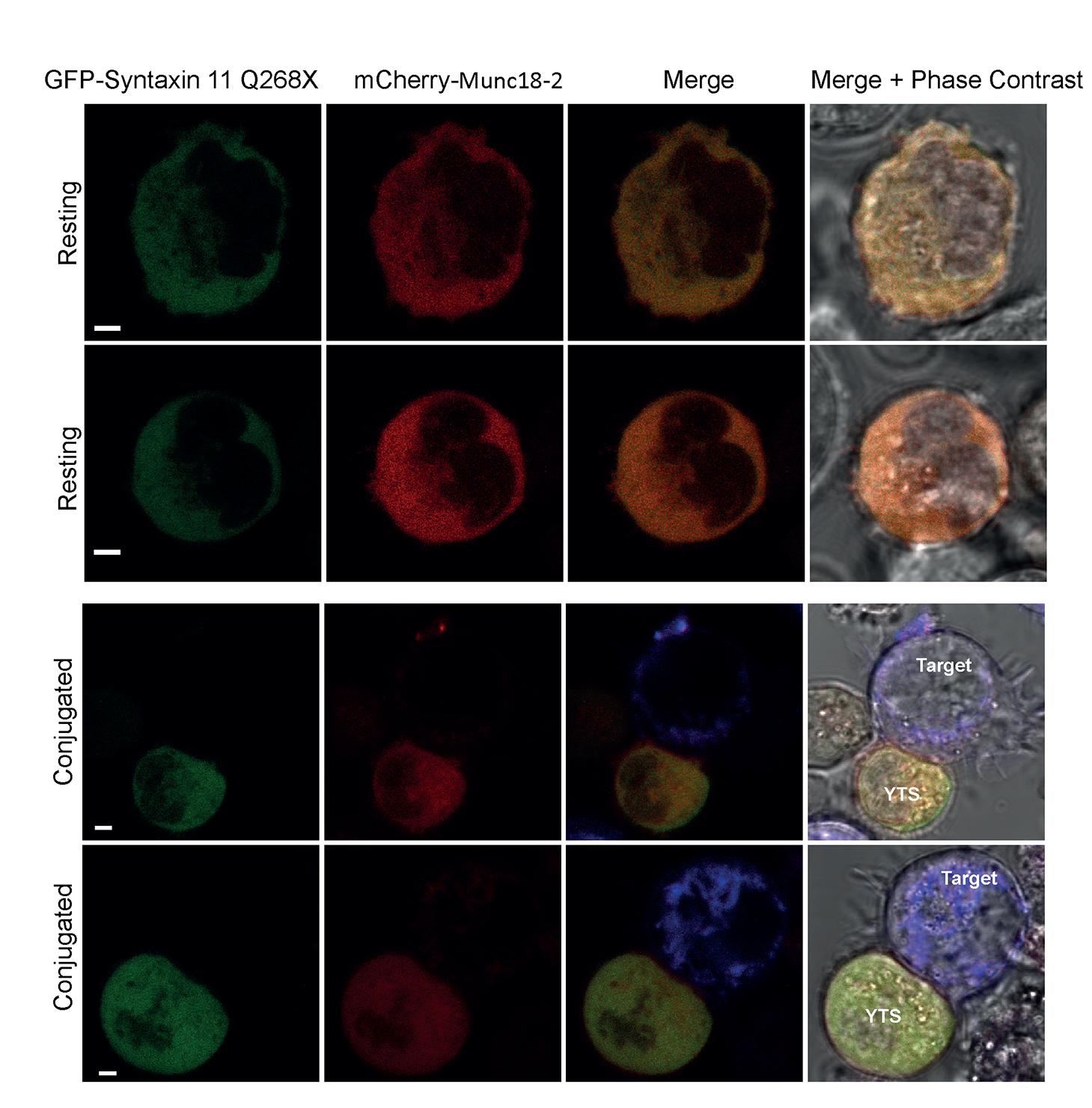

Supplement: Figure S8 — Additional images of the localization of mCherry-Munc18-2 in YTS NK cells co-transfected with GFP-syntaxin 11 Q268X. mCherry-Munc18-2 was co-transfected with GFP-syntaxin 11 Q268X. YTS cells were then imaged in the absence of target cells (Resting) or conjugated to 721.221 target cells pre-stained with Cell Trace Far Red (blue in the merge image panels). Cells were imaged using a Zeiss LSM700 laser scanning confocal microscope. Scale bars 5 µ µm. (TIF) [file pone.0098900.s008.tif]

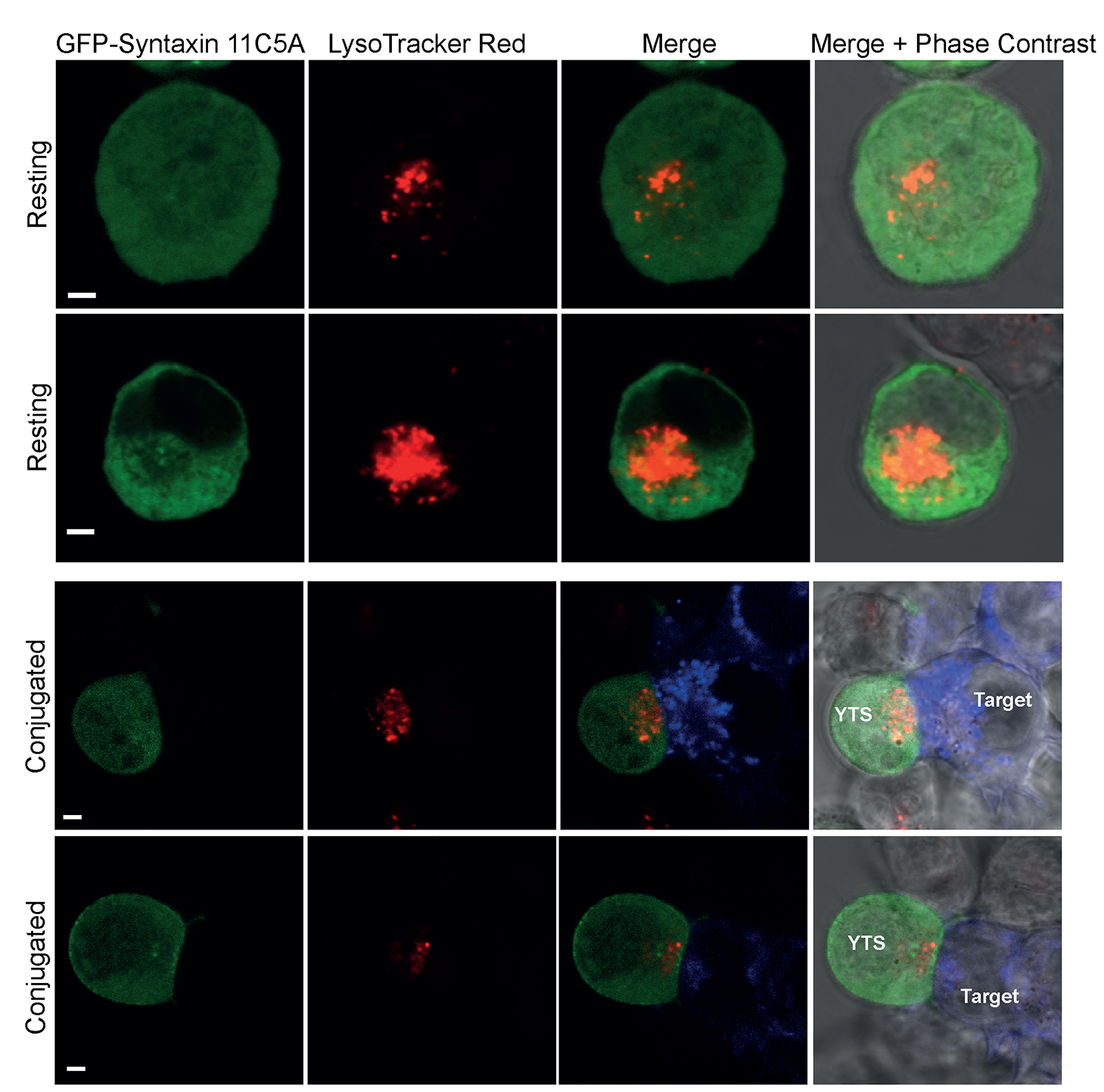

Supplement: Figure S9 — Additional images of the localization of GFP-syntaxin 11 C5A in YTS NK cells. YTS cells expressing GFP-syntaxin 11C5A were then imaged in the absence of target cells (resting) or conjugated to 721.221 cells pre-stained with Cell Trace (blue in the merge image panels). Cells were imaged using a Zeiss LSM700 laser scanning confocal microscope. Scale bars 5 µm. (TIF) [file pone.0098900.s009.tif]
